# ﻿A new microendemic gecko from the small forest fragments of south-eastern Madagascar (Squamata, Gekkonidae, *Paragehyra*)

**DOI:** 10.3897/zookeys.1240.151016

**Published:** 2025-06-02

**Authors:** Francesco Belluardo, Costanza Piccoli, Javier Lobón-Rovira, Ivo Oliveira Alves, Malalatiana Rasoazanany, Franco Andreone, Gonçalo M. Rosa, Angelica Crottini

**Affiliations:** 1 EnviXLab, Department of Biosciences and Territory, University of Molise, Contrada Fonte Lappone s.n.c., 86090 Pesche, Italy; 2 CIBIO, Centro de Investigação em Biodiversidade e Recursos Genéticos, InBIO Laboratório Associado, Universidade do Porto, Campus de Vairão, Rua Padre Armando Quintas, 4485-661 Vairão, Portugal; 3 Departamento de Biologia, Faculdade de Ciências, Universidade do Porto, Rua do Campo Alegre s/n, 4169-007 Porto, Portugal; 4 BIOPOLIS Program in Genomics, Biodiversity and Land Planning, CIBIO, Campus de Vairão, Rua Padre Armando Quintas, 4485-661 Vairão, Portugal; 5 Departamento de Biologia Animal, Faculdade de Ciências, Universidade de Lisboa, Campo Grande, 1749-016 Lisboa, Portugal; 6 Mention Zoologie et Biodiversité Animale, Domaine Sciences et Technologieìiscussions, Université d’Antananarivo, B.P. 906, 101 Antananarivo, Madagascar; 7 Museo Regionale di Scienze Naturali, Via G. Giolitti 36, 10123 Torino, Italy; 8 IMIB Biodiversity Research Institute (CSIC, Universidad de Oviedo, Principality of Asturias), c/ Gonzalo Gutiérrez Quirós, 33600 Mieres, Spain; 9 Institute of Zoology, Zoological Society of London, Outer Circle, Regent’s Park NW1, 4RY London, UK; 10 Centre for Ecology, Evolution and Environmental Changes (CE3C), Faculdade de Ciências, Universidade de Lisboa, Campo Grande, 1749–016 Lisboa, Portugal; 11 Department of Biology, University of Florence, Via Madonna del Piano 6, I-50019 Sesto Fiorentino, Italy

**Keywords:** Community-based management, deforestation, integrative taxonomy, mitochondrial DNA, morphology, nuclear DNA, reptiles

## Abstract

Historically, herpetological research in Madagascar has largely overlooked small forest fragments outside the country protected area network. Despite substantial declines in species diversity compared to large continuous forests, these fragments continue to sustain diverse herpetological communities and frequently harbour microendemic species. We describe a new gecko belonging to the genus *Paragehyra*, apparently microendemic to small and isolated forest fragments surrounding the Andringitra Massif in south-eastern Madagascar. *Paragehyratsaranoro***sp. nov.** is different from its congeneric species based on genetic distances in mitochondrial markers (16S and COI), phylogenetic position, and the lack of haplotype sharing at one nuclear locus (POMC). The new species is also distinguishable from its congeners based on a combination of 14 morphological characters. New genetic and morphological data are also provided for the sympatric *P.felicitae* and we propose a new assessment of its conservation status within the IUCN Red List. *Paragehyratsaranoro***sp. nov.** and *P.felicitae* are mostly found in forest fragments managed by local communities (community-managed reserves) outside legally protected areas. This study highlights the importance of community-based management for the conservation of local herpetofauna, particularly in regions heavily impacted by anthropogenic pressure and largely unsuitable for forest-dwelling species. The findings emphasise the importance of conducting research on small forest fragments, as they are essential for completing the inventory of Malagasy herpetofauna.

## ﻿Introduction

Madagascar is recognised as a global hotspot of reptile diversity (Roll et al. 2017). Currently, the island hosts 439 nominal species of native terrestrial reptiles, with 98% of these being endemic (Antonelli et al. 2022; Glaw et al. 2022; Raselimanana and Vences 2022; Uetz et al. 2025) and approximately one-third being microendemic (i.e., with distributional ranges lower than 1,000 km^2^; sensu Brown et al. 2016). Since 1990 the rate of reptile species descriptions in Madagascar has surged, with ca 40% of native species formally described, although the likely significant level of undescribed diversity (Nagy et al. 2012; Moura and Jetz 2021; Antonelli et al. 2022; Glaw et al. 2022). Such accelerated rate results from the combination of increased fieldwork efforts for species inventories and the use of molecular techniques for species identification (associated with a constantly increasing reference database), which together have expedited the identification of candidate species (sensu Vieites et al. 2009) (Yoder et al. 2005; D’Cruze et al. 2009; Nagy et al. 2012; Glaw et al. 2022; Vences et al. 2022a). The application of a taxonomic approach integrating multiple lines of evidence for species delimitation facilitates rapid and reliable evaluations of the taxonomic status of candidate species, aiding in their formal description (Vieites et al. 2009; Padial et al. 2010; Carné and Vieites 2024; Miralles et al. 2024).

The process of biodiversity cataloguing and description is essential in a period of global biodiversity crisis, as many lineages that are unknown to science might have gone or will go extinct before being identified and formally protected (Giam et al. 2012; Primack 2014; Dijkstra 2016; Chapple et al. 2021). In the case of Madagascar, deforestation is among the main threats to biodiversity, with almost half of the forest cover that was estimated in 1953 to have been lost since then, and many of the remaining forests that suffer from severe fragmentation and degradation (see Lehtinen and Ramanamanjato 2006; Irwin et al. 2010; Jenkins et al. 2014; Nopper et al. 2018; Vieilledent et al. 2018; Crottini et al. 2022; Ralimanana et al. 2022). In Madagascar, herpetological research has historically focused on protected areas and more pristine forests (D’Cruze et al. 2009; Vences et al. 2022a). Despite small and heavily impacted forest fragments tend to support poorer herpetological communities compared to larger, continuous forests, recent inventories have nonetheless led to the identification of several new candidate species (e.g., Lehtinen and Ramanamanjato 2006; Irwin et al. 2010; Crottini et al. 2011a, 2014; Durkin et al. 2011; Belluardo et al. 2021a). Many of these candidate species have since been formally described (e.g., Gehring et al. 2010; Crottini et al. 2011b, 2012a, 2015; Prötzel et al. 2018).

Geckos are the most species-rich squamate group in Madagascar (Antonelli et al. 2022; Bauer et al. 2022; Uetz et al. 2025). They are divided into 11 genera belonging to the family Gekkonidae Oppel, 1811, although they represent multiple clades with independent origins and colonisation histories (Crottini et al. 2012b; Gamble et al. 2015; Zheng and Wiens 2016; Antonelli et al. 2022; Bauer et al. 2022). The genus *Paragehyra* Angel, 1929 is endemic to Madagascar and currently comprises four nominal species. These animals are mainly nocturnal and rupicolous, being mostly found on large boulders, cliffs, and caves, often associated with water courses (Nussbaum and Raxworthy 1994; Glaw and Vences 2007; Crottini et al. 2015). *Paragehyraaustini* Crottini, Harris, Miralles, Glaw, Jenkins, Randrianantoandro, Bauer & Vences, 2015 and *P.gabriellae* Nussbaum & Raxworthy, 1994 are distributed in the extreme south-east of Madagascar. *Paragehyraaustini* is known from a single locality of montane rainforest in the western slopes of the Andohahela Massif, whereas *P.gabriellae* is found in lowland rainforests between the eastern slopes of the Andohahela Massif to the west and the coastal mountain ranges of Vohimena and Anosyenne to the east and south (Nussbaum and Raxworthy 1994; Crottini et al. 2015). *Paragehyrapetiti* Angel, 1929 is distributed in south-western Madagascar, close to the city of Toliara, one of the most arid regions of the island (Nussbaum and Raxworthy 1994). The few specimens known for this species were found in a calcareous area surrounded by arid thornbush vegetation within an alluvial plain next to the coast (Nussbaum and Raxworthy 1994; Glaw and Schmidt 2003; Crottini et al. 2015). *Paragehyrafelicitae* Crottini, Harris, Miralles, Glaw, Jenkins, Randrianantoandro, Bauer & Vences, 2015 is known from two fragments of semi-deciduous dry forest located in the western part of the region surrounding the Andringitra Massif, close to the town of Ambalavao (south-eastern Madagascar) (Crottini et al. 2015; Belluardo et al. 2021a). Finally, another population of *Paragehyra* (P.sp. aff.petiti 1 sensu Crottini et al. 2015) is known from Tsingy de Bemaraha in western Madagascar (Glaw and Vences 2007; Bora et al. 2010; Crottini et al. 2015). Although morphological data suggest it might represent a new species to science, it was not possible to fully evaluate the taxonomic status of this population due to the lack of genetic data and available specimens (Crottini et al. 2015).

The genus *Paragehyra* has been poorly studied for a long time, with the holotype of the type species *P.petiti* being the only known specimen until ca 30 years ago (Angel 1929; Nussbaum and Raxworthy 1994). When Nussbaum and Raxworthy (1994) described the second species *P.gabriellae*, they also re-diagnosed the genus by providing two derived morphological characters to discriminate *Paragehyra* from the remaining gekkonids. They also defined nine interspecific morphological diagnostic traits, which they used to re-described *P.petiti*. Approximately 20 years later, Crottini et al. (2015) expanded on this work by describing *P.austini* and *P.felicitae* using an integrative taxonomic approach combining morphological and molecular evidence. They also defined five additional diagnostic morphological characters to discriminate between congeneric species and produced molecular data from all nominal species. This data was analysed within a phylogenetic framework, providing the first species-level phylogenetic hypothesis for the genus (Crottini et al. 2015).

**Figure 1.**
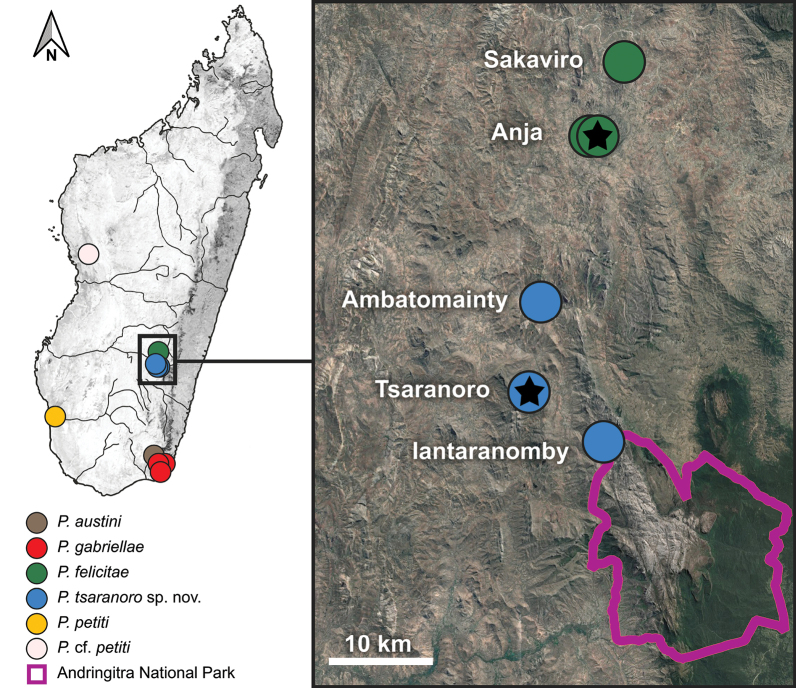
Distribution of the genus *Paragehyra* in Madagascar, with a focus on the Andringitra Massif region, where *P.tsaranoro* sp. nov. and *P.felicitae* occur in sympatry. Iantaranomby is within the borders of Andringitra National Park. Star symbols denote the type localities of the two species. Coordinates of sampling localities are provided in Suppl. material 1. Map created using the Free and Open Source QGIS (Map data ©2015 Google). Species names anticipate the taxonomic decisions proposed in this study.

**Figure 2.**
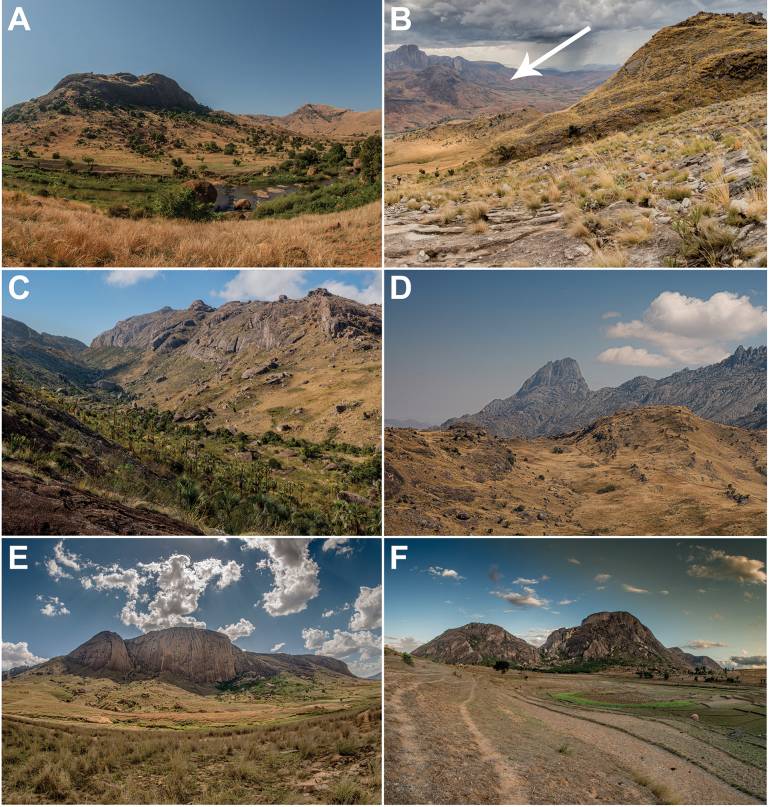
Sampling sites of *P.tsaranoro* sp. nov. (**A**−**C**) and *P.felicitae* (**E, F**) **A** Ambatomainty **B** western slopes of the Andringitra Massif with Tsaranoro forest in the distance indicated with an arrow **C** Iantaranomby (Andringitra National Park) **D** western side of Andringitra Massif **E** Sakaviro **F** Anja. Photographs by JL-R. Species names anticipate the taxonomic decisions proposed in this study.

In this study, we formally describe the lineage of *Paragehyra* previously reported as P.sp. aff.felicitae “Tsaranoro” in Belluardo et al. (2021a), and only recently discovered during a herpetological inventory conducted in the region surrounding the Andringitra Massif (Fig. 1; Belluardo et al. 2021a). The new species was found in the western part of the region, ca 20 km south of the type locality of *P.felicitae*. While Andringitra National Park (Andringitra NP) protects most of the Massif, the western portion of the region is not included in the national network of protected areas and has been heavily impacted by deforestation, with a few remaining and isolated small forest fragments embedded in a human-dominated landscape (Fig. 2; Crottini et al. 2015; Gould and Andrianomena 2015; Gould and Gabriel 2015; Goodman et al. 2018). These remnant forest patches sustain diverse and, in some case, unique herpetological communities, often including microendemic species (Crottini et al. 2011b, 2012a, 2015; Belluardo et al. 2021a; Piccoli et al. 2023). Over the last years, some of these fragments became community-managed reserves, reflecting a shift in Madagascar’s environmental policy toward transferring natural resource management from the central government to local communities. This approach aims to support sustainable livelihoods, enhance local biodiversity conservation, and complement the conservation role of the national protected areas network (Raik 2007; Nopper et al. 2017).

We integrate morphological and molecular data to support the description of P.sp. aff.felicitae “Tsaranoro”. Additionally, we provide new data on the distribution and genetic and morphological variation of *P.felicitae* and propose a conservation status assessment for *P.felicitae* and the new species described here within the IUCN Red List.

## ﻿Materials and methods

This study follows the design outlined by Crottini et al. (2015), expanding the morphological and molecular datasets of the genus *Paragehyra* with samples and specimens from new localities. We conducted new morphological and molecular comparisons, adhering to the same procedures described in that study.

### ﻿Sample codes abbreviations

Extraction number of analysed samples were coded as follows: Angelica Crottini’s extraction codes (AC; ACP), Aurélien Miralles’ extraction code (AM). Field collection numbers were coded as follows: Angelica Crottini Zoological Collection (ACZC), Angelica Crottini Zoological Collection Voucher (ACZCV), Frank Glaw’s field series (FGMV; FGZC), Zoological Collection Miguel Vences (ZCMV), Aurélien Miralles’ field series (MIRZC), and Richard K. B. Jenkins’ field series (RBJ). Museum collection numbers were coded as follows: Kyoto University Museum, Kyoto, Japan (**KUZ**), Mention Zoologie et Biodiversité Animale, Université d’Antananarivo, Antananarivo, Madagascar (**UADBA**), and Zoologische Staatssammlung, Munich, Germany (**ZSM**).

### ﻿Sampling and voucher collection

The specimens and tissue samples included in this study were sampled during a herpetological inventory conducted in the region of the Andringitra Massif in November and December 2018 (see Belluardo et al. 2021a). The area is dominated by the mountain chain (protected by Andringitra NP), which divides the region into an eastern part characterised by the presence of humid climatic conditions and rainforest and a western part with drier conditions and the occurrence of semi-deciduous dry forest (Goodman 1996; Goodman et al. 2018). The study area is located on the western side of the region (Fig. 1), which hosts, especially outside Andringitra Massif and Andringitra NP, several small and isolated forest fragments composed of semi-deciduous and southern dry-adapted rupicolous vegetation located at the base of small granitic peaks at ca 900 m a.s.l. Three among the largest fragments are community-managed reserves: Tsaranoro (46 ha), Anja (36 ha), and Sakaviro (14 ha) (Fig. 2; Gould and Andrianomena 2015; Gould and Gabriel 2015; Gabriel et al. 2018; Belluardo et al. 2021a).

**Figure 3.**
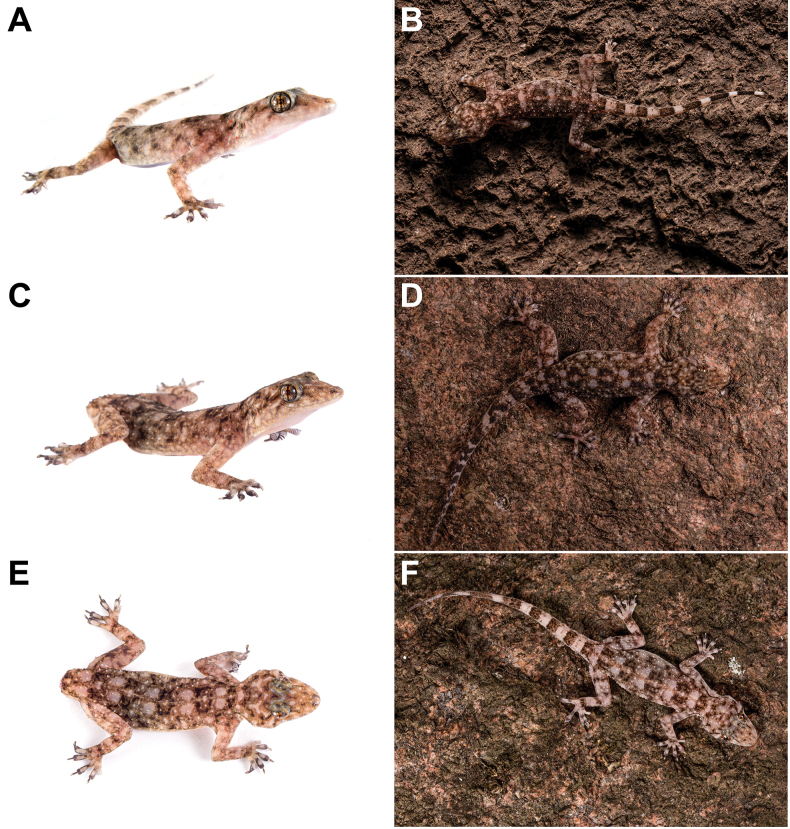
Photographs of *Paragehyratsaranoro* sp. nov. **A** holotype ZSM 11/2023 (ACZCV765) from Tsaranoro **B** ACZC10947 from Tsaranoro (voucher not collected) **C**, **E** paratype ZSM 10/2023 (ACZCV600) from Iantaranomby (western slopes of Andringitra National Park) **D** ACZC10946 from Tsaranoro (voucher not collected) **F** ACZC10951 from Tsaranoro (voucher not collected). See Suppl. material 1 for sampling information and associated sequences available for these individuals. Photographs by JL-R. Species names anticipate the taxonomic decisions proposed in this study.

We collected tissue samples from 15 individuals of *P.felicitae* at two localities (Anja and Sakaviro) and 15 individuals of P.sp. aff.felicitae “Tsaranoro” at three localities (Tsaranoro, Ambatomainty, and Iantaranomby) (Figs 1–3; Suppl. material 1). Animals were caught by hand during opportunistic searches. Each individual was photographed, and the collection locality was recorded with a GPS receiver (Suppl. material 1). We clipped tail tips from each animal (or a toe in case of voucher specimens; see below) and stored them in 96% ethanol for molecular analyses. Four individuals of *P.felicitae* and seven individuals of P.sp. aff.felicitae “Tsaranoro” were euthanised with an intracoelomic injection of a saturated solution of MS222 and kept as vouchers (Suppl. material 1). Vouchers were fixed in 96% ethanol and kept in 70% ethanol for long-term storage and deposited in the institutional collections of ZSM and UADBA.

### ﻿Morphological measurements

Morphological characters of the voucher specimens were inspected by FB and confirmed by AC, CP, and IOA. FB took morphometric measurements with a digital calliper to the nearest 0.1 mm (Table 1; Suppl. material 2). The scheme of the species description and the definition of morphological characters follow Nussbaum and Raxworthy (1994) and Crottini et al. (2015), and the corresponding abbreviations are the same used in Crottini et al. (2015): **HT**, holotype; **PT**, paratype; **M**, male; **F**, female; **J**, juvenile; **SVL**, snout-vent length; **TAL**, tail length; **TBW**, tail base width; **BW**, body width; **HL**, head length; **HW**, head width; **HD**, head depth; **SnL**, snout length (mouth opening); **ID**, internarial distance; **IOD**, interorbital distance; **ETD**, eye-tympanum distance; **ED**, eye diameter; **EO**, ear opening (horizontal axis); **SAD**, snout-axilla distance (measured from the tip of the snout to the axilla with the forelimb extended laterally); **AGD**, axilla-groin distance; **FL**, forelimb length (measured from the point where the limb attaches to the axilla to the tip of the longest digit); **HiL**, hind limb length (measured from the point where the limb attaches to the groin to the tip of the longest digit); **IN**, internasal scales; **SL**, number of enlarged supralabial scales; **I**, infralabial scales; **ME**, mental scale; **1PM**, dimension of first postmental scales; **2PM**, number of second postmental scales (defined as the enlarged scales in contact with first postmentals frontally and with small chin scales posteriorly); **C**, chin scales (defined as the small scales extending frontally towards the complex of infralabial and postmental scales); **DO**, dorsal scales on body surface; **BT**, body tubercles and number of longitudinal rows of enlarged tubercles on body; **TDL**, tubercles on dorsal surface of limbs; **TT**, tail tubercles; **VE**, ventral scales on body surface; **VET**, ventral scales on distal (tibial) segment of hind limbs; **SC**, subcaudal scales; **SCE**, subcaudal scales enlarged transversely; **PCP**, number of precloacal pores; **SPP**, scales on preaxial-ventral portion of pes between end of tibia and base of digit I; **SS**, subdigital scales between enlarged basal scales and distal pad on digit I of manus and pes; **SLMP**, number of transversely enlarged subdigital lamellae on claw-bearing segment digits II−V of manus and pes; **SSC**, subdigital scales on claw-bearing segment of digits II−V of manus and pes.

**Table 1.** Variation of morphological diagnostic characters of *Paragehyra* specimens. Specimens of *Paragehyratsaranoro* sp. nov. and the new individuals of *P.felicitae* analysed in this study are highlighted in bold, while the remaining specimens are from Crottini et al. (2015). Information on character states provided in the table footer. Species names anticipate the taxonomic decisions proposed in this study.

Species	Catalogue number	Status	Sex	BT	TDL	TT	I	C	VET	SPP	SS	SSC	DO	VE	SC	ME	1PM
*P.austini*	ZSM 0338/2005	PT	F	-	-	-	1 (9/8)	1	2	s7	4-3	6,5,6,6 a; 6,6,6,5 a	s=	s+	s+	2	L 50+
*P.austini*	ZSM 0339/2005	HT	M	-	-	-	1 (8/8)	1	2	s7	3-3	4,3,3,5 a; 4,4,5,6 a	s=	s+	s+	2	L 50+
*P.austini*	ZSM 0340/2005	PT	M	-	-	-	1 (8/9)	1	2	s7	4-3	4,5,6,5 a; 6,7,5,4 a	s=	s+	s+	2	L 50+
*P.gabriellae*	ZSM 0085/2004	_	NA	+ *	-	*	1 (9/8)	2	1	s8	9-7	11,11,10,11 b; 12,11,10,10 b	s=	s+	s+	2	L 50-
*P.gabriellae*	ZSM 0114/2004	_	NA	+ *	-	*	1 (9/8)	2	1	s7	8-7	10,10,10,10 b; 10,9,11,10 b	s=	s+	s+	2	L 50-
*P.gabriellae*	ZSM 0173/2004	_	NA	+ *	-	*	1 (9/8)	3	1	s8	9-9	10,9,9,11 b; 11,10,10,11 b	s=	s+	s+	2	L 50-
*P.gabriellae*	ZSM 0181/2004	_	NA	+ *	-	*	1 (10/9)	2	1	s8	9-8	10,10,10,9 b; 10,10,9,11 b	s=	s+	s+	2	L50
*P.gabriellae*	ZSM 0182/2004	_	NA	+ *	-	*	1(9/8)	2	1	s8	9-10	10,12,11,12 b; 11,12,12,12 b	s=	s+	s+	2	L50
*P.gabriellae*	ZSM 0336/2005	_	NA	+ *	-	*	1 (9/9)	Le 3; R 1	1	s9	9-9	9,10,11,10 b; 10,11,10,10 b	s=	s+	s+	2	L50
*P.gabriellae*	ZSM 0337/2005	_	NA	+ *	-	*	1 (9/9)	Le 3; R 2	1	s7	10-9	10,11,10,11 b; 12,12,11,11 b	s=	s+	s+	2	L50
*P.gabriellae*	ZSM 0335/2005	_	J	+ *	-	NA	1 (10/9)	3	1	s9	9-9	10,10,10,10 b; 12,11,11,11 b	s=	s+	NA	2	L 50-
*P.felicitae*	ZSM 1613/2010	PT	F	+/12	+	+	1 (9/7)	3	3 (6)	s9	3-2	5,5,5,5 c; 5,5,6,5 c	s=	s-	s+	2	L 50+
*P.felicitae*	ZSM 1612/2010	PT	M	+/12	+	+	1 (8/7)	1	3 (6)	l6	3-3	4,5,5,5 c; 6,5,5,5 c	s=	s-	s+	2	L 50+
*P.felicitae*	ZSM 1611/2010	HT	M	+/12	+	+	1 (8/9)	3	3 (6)	l6	3-3	5,5,5,5 c; 6,6,5,5 c	s=	s-	s+	2	L 50+
*P.felicitae*	ZSM 1610/2010	PT	M	+/12	+	+	1 (8/9)	1	3 (6)	l6	2-3	5,5,5,5 c; 5,5,5,5 c	s=	s-	s+	2	L 50+
***P.felicitae***	**ZSM 14/2023 (ACZCV747)**	_	**F**	+/**12**	+	+	**1 (8/7)**	**1**	**3 (7)**	**l7/l8**	**3-3**	**5,5,5,6 c; 4,5,5,4 c**	**s**-	**s**-	**s**+	**1**	**L 50**+
***P.felicitae***	**ZSM 13/2023 (ACZCV522)**	_	**J**	+/**12**	+	+	**1 (8/7)**	**1**	**3 (3)**	**l7/l6**	**3-2**	**5,4,4,4 a; 5,4,5,5 c**	**s**-	**s**-	**s**+	**2**	**L 50**+
***P.tsaranoro* sp. nov.**	**ZSM 10/2023 (ACZCV600)**	**PT**	**F**	+/**12**	+ *	**NA**	**1 (7/6)**	**1**	**3 (6)**	**l5/l6**	**3-2**	**5,5,5,4 a; 4,5,5,6 a**	**s**-	**s**-	**NA**	**2**	**L 50**+
***P.tsaranoro* sp. nov.**	**ZSM 11/2023 (ACZCV765)**	**HT**	**M**	+/**12**	+ *	+	**1 (7/8)**	**Le 1; R 4**	**3 (3)**	**l5/l6**	**3-3**	**5,5,5,5 a; 5,6,5,5 a**	**s**-	**s**-	**s**+	**2**	**L 50**+
***P.tsaranoro* sp. nov.**	**ZSM 12/2023 (ACZCV809)**	_	**J**	+/**12**	+ *	+	**1 (8/8)**	**1**	**3 (4)**	**l6**	**2-3**	**5,5,5,1 a; 5,4,6,5 a**	**s**-	**s**-	**s**+	1*	**L 50**+
*P.petiti*	ZSM 593/2000	_	M	+/10	+ *	+	2 (4/4)	1	3 (5)	l5	3-3	5,5,4,4 c; 4,6,5,5 c	s-	s-	s-	1	L 50+
*P.petiti*	ZSM 594/2000	_	M	+/10	+ *	+	2 (5/5)	1	3 (6)	l5	2-2	4,4,4,4 c; 4,4,4,4 c	s-	s-	s-	1	L 50+
*P.petiti*	ZSM 592/2000	_	F	+/10	+ *	+	2 (6/5)	Le 4; R 1	3 (6)	l5	2-3	4,4,5,4 c; 4,4,4,4 c	s-	s-	s-	1	L 50+
P.cf.petiti	UADBA 28056	_	M	+/10	+	+	1 (6/5)	1	3 (6)	l7	NA	NA	s-	s-	s+	1*	L 50+
P.cf.petiti	UADBA 28038	_	M	+/10£	+	+	1 (6/5)	1	3 (6)	l7	NA	NA	s-	s-	s+	1*	L 50+

Abbreviations not reported in the text: Le, left; R, right. Description of the alternative character states: BT, body tubercles and number of longitudinal rows of enlarged tubercles on body (+, present; -, absent; *, small tubercles not arranged in distinct rows; £, 9 complete rows and 1 truncated row; #, number of rows); TDL, tubercles on dorsal surface of limbs (+, present; -, absent; *, present on hind limbs and only on distal portions on forelimbs); TT, tail tubercles (+, present and organised in transverse rows; -, absent; *, present but not organised in transverse rows); I, infralabial scales (1, decrease in size gradually in posterior direction; 2, first scales markedly larger than remainders; #, number of scales right/left); C, chin scales (1, lateral chin scales extend forward along each side, excluded from contact with first line of infralabials and first postmentals, in contact with second line of infralabials; 2, lateral chin scales extend forward along each side, in contact with first and second infralabials and with first postmentals; 3, lateral chin scales extend forward along each side, excluded from contact with first infralabials, in contact with first postmentals and second infralabials; 4, lateral chin scales extend forward along each side, excluded from contact with first and second infralabials and first postmentals, in contact with third infralabials); VET, ventral scales on distal (tibial) segment of hind limbs (1, normal size compared with scales on the proximal segments of hind limbs; 2, slightly larger; 3, enlarged into plates especially distantly; #, number of plates); SPP, scales on preaxial-ventral portion of pes between end of tibia and base of digit I (s, small; l, large; #, number of scales right/left); SS, subdigital scales between enlarged basal scales and distal pad on digit I of manus and pes (left side) (#, number of scales manus-pes); SSC, subdigital scales on claw-bearing segment of digits II−V of manus and pes (left side) (a, distalmost scale markedly larger; b, numerous small scales increasing gradually in size distantly; c, large scales almost equal in size; #, number of scales); DO, dorsal scales on body surface (s, smooth; k, keeled; =, equal size than ventrals; -, smaller than ventrals); VE, ventral scales on body surface (s, smooth; k, keeled; +, pigmented; -, unpigmented or poorly pigmented); SC, subcaudal scales (s, smooth; k, keeled; +, pigmented; -, unpigmented); ME, mental scale (1, bell-shaped; 2, triangle-shaped; *, modified); 1PM, dimension of first postmental scales (L, large; 50+, in contact for more than the 50% of their length; 50-, in contact for less than the 50% of their length; 50, in contact for the 50% of their length).

### ﻿Molecular procedures

The newly collected voucher specimens included in this study have been molecularly identified at the species level in Belluardo et al. (2021a) based on the inter-specific threshold of genetic distance at the cytochrome oxidase I gene (COI) suggested by Nagy et al. (2012) for geckos (accession numbers MZ285511–MZ285520; Suppl. material 1). In addition, sequences of the 16S gene fragment were used to complement species identification (accession numbers MZ285405–MZ285408; Suppl. material 1; Crottini et al. 2015; Belluardo et al. 2021a).

We expanded the available molecular data by generating new sequences from these specimens and from tissue samples collected from individuals not retained as vouchers (Suppl. material 1). Specifically, we extracted total genomic DNA following a saline solution extraction protocol (Bruford et al. 1992). We amplified one mitochondrial and two nuclear markers: a fragment of the 3’ terminus of the 16S rRNA gene (16S), a fragment of the brain-derived neurotrophic factor gene (BDNF) and a fragment of the pro-opiomelanocortin gene (POMC) (Table 2). Amplifications were performed with a standard PCR protocol in a final reaction volume of 25 µl using 12.5 µl of Milli-Q water, 5 µl of 5X Green GoTaq Flexi Buffer (Promega, Madison, US), 4 µl of MgCl2 (25 mM) (Promega), 1 µl of each primer (10 pM) (Thermo Fisher Scientific, Waltham, US), 0.4 µl of dNTPs (10 mM) (Invitrogen, Waltham, US), 0.1 µl of 5 U/µl GoTaq Flexi DNA Polymerase (Promega), and 1 µl of extracted genomic DNA (see Table 2 for information on thermal profiles and primers). Successfully amplified samples were sequenced with an ABI 3730XL automated sequencer at Macrogen Inc. (Spain). Chromatograms were checked and edited, when necessary, using BIOEDIT 7.2.6 (Hall 1999). Newly generated sequences were deposited in GenBank (PV383205–PV383230; PV389996–PV390006; Suppl. material 1).

**Table 2.** Fragments amplified in this study with information on primer sequences and amplification conditions.

Gene	Primer name	Sequence (5’–3’)	Reference	PCR conditions
16S	F: 16SL	CGCCTGTTTATCAAAAACAT	Palumbi et al. 1991	94 °C (90s), [94 °C (45s), 55 °C (45s), 72 °C (90s)] × 33, 72 °C (600s)
16S	R: 16SH	CCGGTCTGAACTCAGATCACG	Palumbi et al. 1991	
BDNF	F: BDNF DRV F1	ACCATCCTTTTCCTKACTATGG	Vieites et al. 2007	94 °C (120s), [94 °C (20s), 52 °C (45s), 72 °C (120s)] × 39, 72 °C (600s)
BDNF	R: BDNF DRV R1	CTATCTTCCCCTTTTAATGGTC	Vieites et al. 2007	
POMC	F: POMC DRV F1	ATATGTCATGASCCAYTTYCGCTGGAA	Vieites et al. 2007	95 °C (180s), [95 °C (60s), 44 °C (60s), 72 °C (90s)] × 40, 72 °C (600s)
POMC	R: POMC DRV R1	GGCRTTYTTGAAWAGAGTCATTAGWGG	Vieites et al. 2007	

### ﻿Molecular analyses

Crottini et al. (2015) compiled a molecular dataset of the markers 16S, BDNF, POMC, and recombination activating gene 1 (Rag1) with several individuals belonging to all nominal species of the genus *Paragehyra*. We populated these datasets with the new sequences for the same markers (with the exception of Rag1, which was not analysed here; Suppl. material 1). We aligned sequences of each marker with the CLUSTAL W algorithm implemented in BIOEDIT 7.2.6 (Thompson et al. 1994; Hall 1999). Uncorrected pairwise genetic distances (uncorrected *p*-distances) within (between conspecific individuals) and between (averaged over conspecific individuals) species were computed on the 16S alignment with MEGA X 10.0.5 (Kumar et al. 2018). Following the same procedure described for 16S, we computed uncorrected *p*-distances within and between species on an alignment including all COI sequences available in GenBank for the genus *Paragehyra* (Suppl. material 1).

We inferred haplotype networks of the nuclear makers BDNF and POMC. We first trimmed all sequences to the same length (all sequences have been deposited to GenBank in their original length). The BDNF sequence of ACP5938 (P.sp. aff.felicitae “Tsaranoro”) has been deposited (PV390004), although it was excluded from the haplotype network analyses because it was much shorter than the others. We phased the alignments of the BDNF and POMC markers with the PHASE algorithm (Stephens et al. 2001; Stephens and Donnelly 2003) implemented in DNASP 6.12.03 (Rozas et al. 2017), setting 1,000 iterations, 10% burn-in and replicating the analyses three ×, each analysis with a different starting seed value. We then inferred maximum likelihood (ML) trees from the phased alignments in MEGA X 10.0.5 (Kumar et al. 2018) setting Jukes-Cantor as substitution model. Haplotype networks were then computed for each marker on the phased alignments and the inferred ML trees with HAPLOTYPE VIEWER (written by G. B. Ewing; http://www.cibiv.at/~greg/haploviewer.shtml) to build a network from the tree topology following the methodological approach described in Salzburger et al. (2011).

We performed a phylogenetic analysis on the 16S alignment using PIPELOGENY on R 4.0.2 (Muñoz-Pajares et al. 2020; R Core Team 2022). This pipeline facilitates the automated preparation of the input files for phylogenetic tools, which were subsequently executed in their respective standalone software applications. We used JMODELTEST 2.1.10 (Darriba et al. 2012) on the CIPRES Science Gateway portal (Miller et al. 2010) to find the best model of sequence evolution. We inferred the phylogeny with MrBayes 3.2.7 (Ronquist et al. 2012), available on the CIPRES portal, setting two parallel runs of 20-million generations, each consisting of four Markov Chain Monte Carlo chains. We set a sampling frequency of the chain at the 1,000^th^ generation and a 40% burn-in. We evaluated the posterior distributions of each prior and the convergence of the two runs with TRACER 1.7.1 based on a minimum threshold of 200 Effective Sampling Size (Rambaut et al. 2018). The 50%-majority rule consensus tree was visualised with FIGTREE 1.4.4 (Rambaut 2009). We used the species *Eublepharismacularius* (Blyth, 1854), *Geckolepistypica* Grandidier, 1867, *Blaesodactylusantongilensis* (Böhme & Meier, 1980), *Phelsumalineata* Gray, 1842, and *Paroedurastumpffi* (Boettger, 1879) as outgroups in our phylogenetic analysis. *Eublepharismacularius* was set as the primary outgroup in MrBayes analysis (Suppl. material 1).

## ﻿Results

### ﻿Justification for species delimitation

Following the integration by congruence approach (Padial et al. 2010) in defining species as independent evolutionary lineages based on at least two independent lines of evidence supporting their distinctness, our results support the hypothesis that the candidate species P.sp. aff.felicitae “Tsaranoro” is distinct from all the other species of the genus *Paragehyra* and thus represents a new species to science. In anticipation of our taxonomic treatment, from now on we will use the newly proposed name for the new taxon even before the taxonomic section.

**Figure 4.**
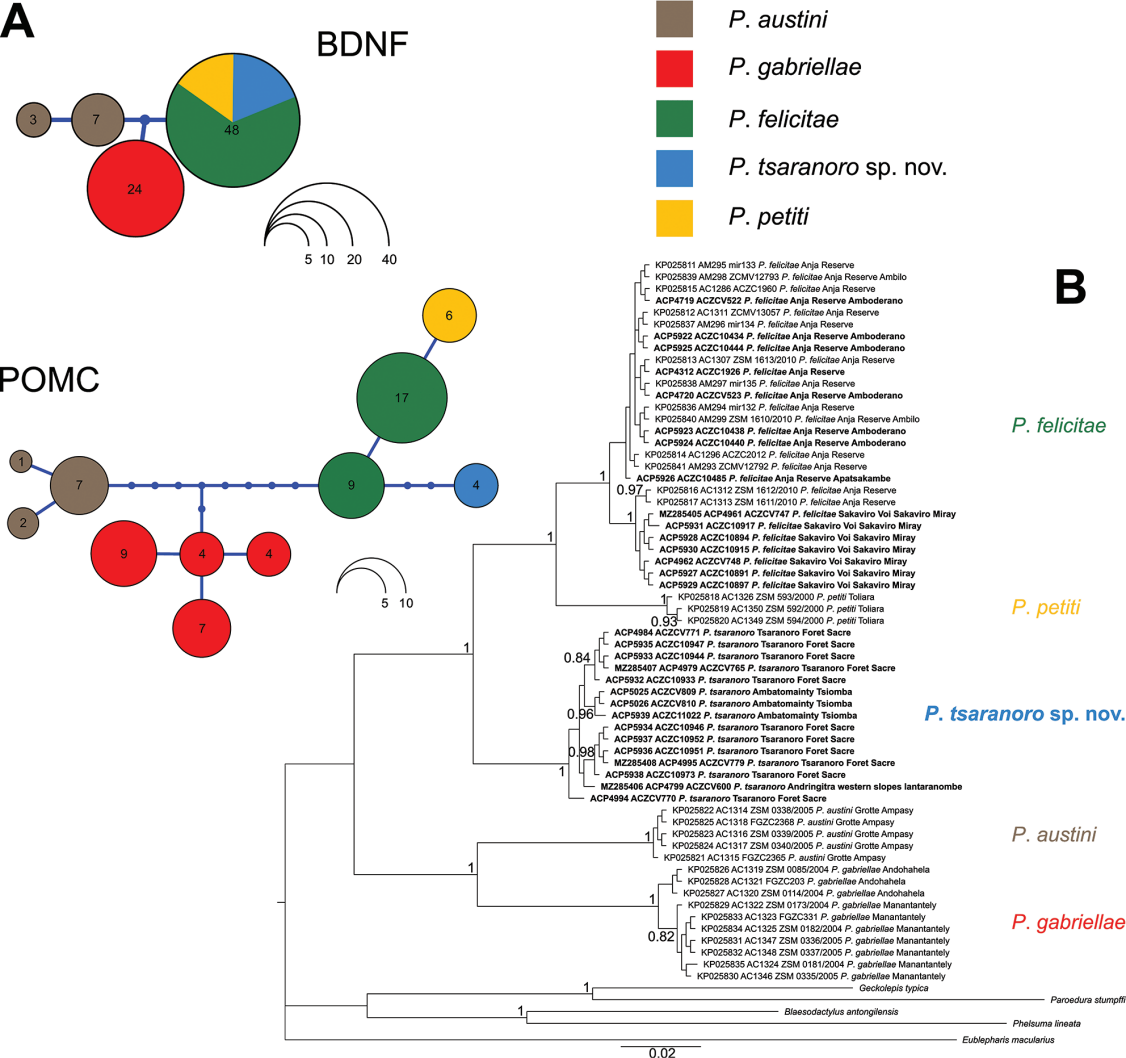
Haplotype networks of nuclear markers and phylogenetic hypothesis for the mitochondrial 16S gene fragment **A** haplotype network reconstructions of BDNF and POMC markers (alignments of 533 bp with 41 individuals and 452 bp with 35 individuals, respectively) of the genus *Paragehyra*. Circles represent haplotypes and report the number of phased sequences assigned to each of them. Dots between circles represent unsampled or extinct haplotypes, whereas segments between dots are the number of reconstructed mutational steps between haplotypes **B**16S phylogenetic tree (50%-majority rule consensus tree) of the genus *Paragehyra* inferred with MrBayes. Posterior Probability values are shown before the respective nodes, and values below 0.80 are not reported. Newly generated sequences are highlighted in bold. Datasets are described in Suppl. material 1.

As a first line of evidence, *P.tsaranoro* sp. nov. resulted as a monophyletic mitochondrial lineage (with full statistical support; Fig. 4B). This lineage showed a range of uncorrected *p*-distances at the 16S and COI gene fragments which were higher than the lowest value observed between two of the currently accepted species of the genus *Paragehyra* (i.e., the distance between *P.felicitae* and *P.petiti*; Table 3). In addition, *P.tsaranoro* sp. nov. shows uncorrected *p*-distances at the COI gene from all the other species for which we had comparative material higher than the standard inter-specific threshold in Malagasy geckos at this marker (i.e., 13.3%) (Table 3; Nagy et al. 2012).

**Table 3.** Uncorrected *p*-distances (16S and COI genes) expressed in percentage within (in bold along the diagonal) and between (below the diagonal) species of the genus *Paragehyra*. The number of individuals used to compute distances are shown in the first column after species names. Distances were computed with MEGA X 10.0.5 (Kumar et al. 2018). Within species uncorrected *p*-distances of the COI gene could not be computed for *P.petiti* and *P.austini* as one individual was available for each of them.

	*P.felicitae*	*P.tsaranoro* sp. nov.	*P.petiti*	*P.austini*	*P.gabriellae*
**16S**					
*P.felicitae* (28)	**0.48**				
*P.tsaranoro* sp. nov. (15)	6.49	**0.36**			
*P.petiti* (3)	5.10	7.46	**0.13**		
*P.austini* (5)	13.95	12.67	13.70	**0.00**	
*P.gabriellae* (10)	13.53	12.93	14.40	9.70	**0.34**
**COI**					
*P.felicitae* (5)	**2.55**				–
*P.tsaranoro* sp. nov. (5)	15.67	**1.28**			–
*P.petiti* (1)	11.03	17.10	**NA**		–
*P.austini* (1)	20.95	20.78	18.98	**NA**	–

*Paragehyratsaranoro* sp. nov. did not share POMC haplotypes with its congeneric species (Fig. 4A). Considering the differences in the inheritance of mitochondrial and nuclear markers and the absence of recombination between them, the concordance between independent markers in supporting the distinctness of this lineage represents another line of evidence (Avise and Ball 1990; Rakotoarison et al. 2017).

**Figure 5.**
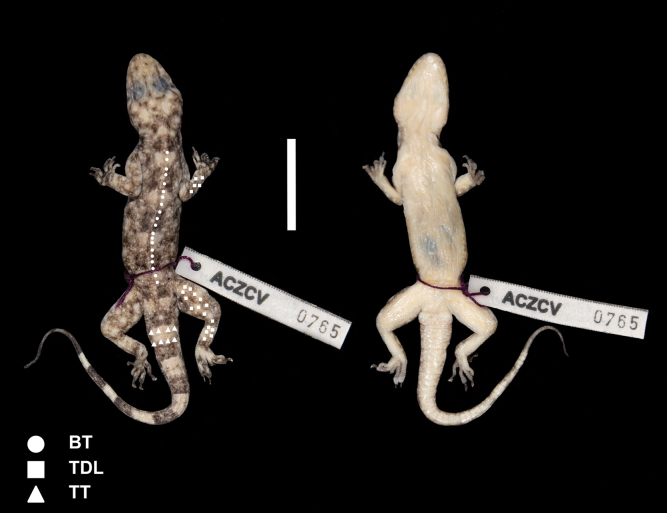
Dorsal and ventral views of *Paragehyratsaranoro* sp. nov. holotype ZSM 11/2023 (ACZCV765). A few diagnostic characters are visible. In the dorsal view: 12 distinct longitudinal rows of enlarged tubercles on body surface (BT), enlarged tubercles on the entire surface of hind limbs and on distal dorsal portions of forelimbs (character TDL), and enlarged tubercles organised in transversal rows that encircle dorsolateral tail surface (character TT). In the ventral view: smooth unpigmented or poorly pigmented scales on body surface (character VE), and smooth and pigmented subcaudal scales (character SC). Photographs by CP. Scale bar: 2 cm.

**Figure 6.**
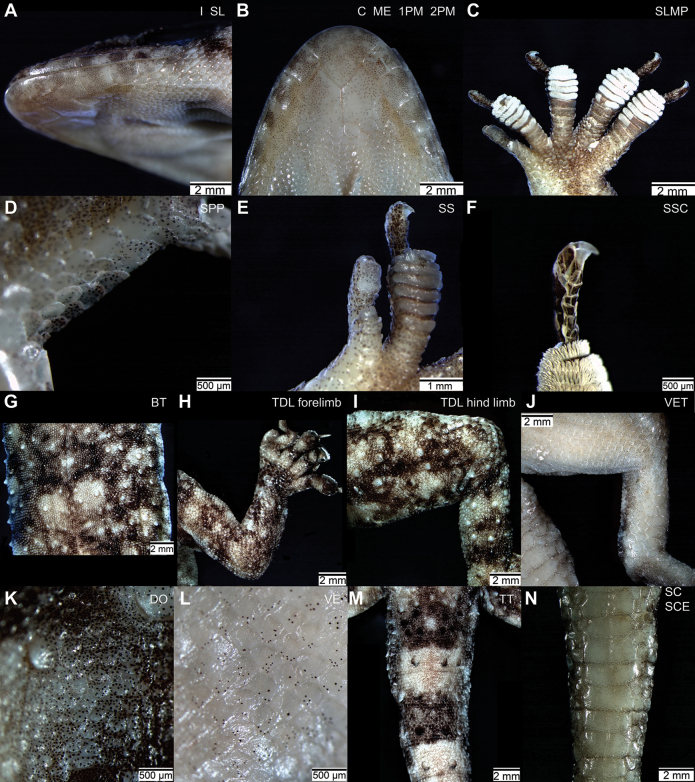
Diagnostic morphological characters of *Paragehyratsaranoro* sp. nov. Part of non-diagnostic characters are also shown (SL, 2PM, SLMP, and SCE; Suppl. material 2), but state descriptions are provided only for diagnostic characters and non-diagnostic SLMP (Tables 1, 4). Pictures taken from the holotype ZSM 11/2023 (ACZCV765) with the exception of **C, E, G−I**, taken from the paratype ZSM 10/2023 (ACZCV600) **A** I, 6−8 enlarged infralabials gradually decreasing in size posteriorly **B**ME, triangle-shaped mental scale; 1PM, large first postmental scales in contact for more than the 50% of their length; **C**, lateral chin scales extend forward along each side, excluded from contact with first line of infralabials and first postmentals and in contact with second line of infralabials C SLMP, 8, 8, 9, 8 transversely enlarged subdigital lamellae on claw-bearing segment digits II−V (left manus in the picture) **D**SPP, five or six large scales on preaxial ventral portion of pes between end of tibia and base of digit I (right pes in the picture) **E**SS, two or three small subdigital scales between enlarged basal scales and distal pad on digit I of manus and pes (left manus in the picture) **F**SSC, 4−6 subdigital scales on claw-bearing segment of digits II−V of manus and pes with the distalmost scale markedly larger than the others (digit III of left pes in the picture) **G**BT, 12 distinct longitudinal rows of enlarged tubercles on the body dorsolateral surface **H**, I TDL, enlarged tubercles on the entire dorsal surface of hind limbs and on distal dorsal portions of forelimbs **J**VET, 3−6 ventral scales on the distal (tibial) segments of hind limbs enlarged into plates especially in the most distal part **K**DO, smooth dorsal scales on body surface smaller than ventral scales **L**VE, smooth unpigmented or poorly pigmented ventral scales on body surface **M**TT, enlarged tubercles organised in transversal rows that encircle tail dorsolateral surface **N**SC, smooth and pigmented subcaudal scales.

Finally, the inspection of the inter-specific morphological characters of the genus *Paragehyra* (Nussbaum and Raxworthy 1994; Crottini et al. 2015) enabled to discriminate *P.tsaranoro* sp. nov. from all congeneric species, therefore, confirming its distinctness also at the morphological level (Table 4; Figs 3, 5, 6). In the following paragraphs, we provide a detailed description of the diagnosis of the new species relative to the remaining species of the genus *Paragehyra* based on molecular and morphological data.

**Table 4.** Morphological diagnostic characters of the genus *Paragehyra*. In bold are different character states relative to *P.tsaranoro* sp. nov. The state definition of characters C, VET, SPP, DO, and ME of *P.felicitae* has been updated relative to the species description (Crottini et al. 2015) following the morphological inspection of the two recently collected voucher specimens (Table 1, Suppl. material 1). See Morphological measurements for character definitions.

Character	*P.tsaranoro* sp. nov.	*P.felicitae*	*P.petiti*	*P.gabriellae*	*P.austini*
BT	12 longitudinal rows of enlarged tubercles on dorsolateral surface of body	12 longitudinal rows of enlarged tubercles on dorsolateral surface of body	**10 longitudinal rows of enlarged tubercles on dorsolateral surface of body**	**Small tubercles not arranged in distinct rows**	**No tubercles on dorsolateral surface of body**
TDL	Presence of enlarged tubercles on the entire dorsal surface of hind limbs and on distal dorsal segments of forelimbs	**Presence of enlarged tubercles on dorsal surface of limbs**	Presence of enlarged tubercles on the entire dorsal surface of hind limbs and on distal dorsal segments of forelimbs	**Absence of enlarged tubercles on dorsal surface of limbs**	**Absence of enlarged tubercles on dorsal surface of limbs**
TT	Transverse rows of enlarged tubercles encircle dorsolateral surface of tail	Transverse rows of enlarged tubercles encircle dorsolateral surface of tail	Transverse rows of enlarged tubercles encircle dorsolateral surface of tail	**Presence of enlarged tubercles on tail not organised in transverse rows**	**Absence of tubercles on dorsolateral surface of tail**
I	6−8 enlarged infralabials diminish gradually in size posteriorly	7−9 enlarged infralabials diminish gradually in size posteriorly	**First 4−6 infralabials much larger than remainders**	8−10 enlarged infralabials diminish gradually in size posteriorly	8 or 9 enlarged infralabials diminish gradually in size posteriorly
C	Lateral chin scales extend forward along each side, excluded from contact with first infralabials and first postmentals, in contact with second infralabials	**Lateral chin scales extend forward along each side, excluded from contact with first line of infralabials and first postmentals, in contact with second line of infralabials or lateral chin scales extend forward along each side, excluded from contact with first infralabials, in contact with first postmentals and second infralabials**	Lateral chin scales extend forward along each side, excluded from contact with first infralabials and first postmentals, in contact with second infralabials	**Lateral chin scales extend forward along each side in contact with first and second infralabials and with first postmentals or lateral chin scales excluded from contact with first infralabials, in contact with first postmentals and second infralabials**	Lateral chin scales extend forward along each side, excluded from contact with first infralabials and first postmentals, in contact with second infralabials
VET	3−6 ventral scales on distal (tibial) segment of hind limb enlarged into plates, especially distally	6−7 ventral scales on distal (tibial) segment of hind limb enlarged into plates, especially distally	5 or 6 enlarged ventral scales on distal (tibial) segment of hind limb	**Ventral scales on distal (tibial) segment of hind limb of normal size compared with scales on the proximal segments of hind limbs**	**Ventral scales on distal (tibial) segment of hind limb slightly larger compared with scales on the proximal segments of hind limbs**
SPP	5 or 6 large scales on preaxial-ventral portion of pes between end of tibia and base of digit I	**Generally 6−9 large scales on preaxial-ventral portion of pes between end of tibia and base of digit I**	5 large scales on preaxial-ventral portion of pes between end of tibia and base of digit I	**7−9 small scales along preaxial-ventral border of pes between end of tibia and base of digit I**	**Generally 7 small scales on preaxial-ventral portion of pes between end of tibia and base of digit I**
SS	2 or 3 small subdigital scales between enlarged basal scales and terminal distal pad and digit I of manus and pes	2 or 3 small subdigital scales between enlarged basal scales and terminal distal pad on digit I of manus and pes	2 or 3 small subdigital scales between enlarged basal scales and terminal distal pad on digit I of manus and pes	**7−10 small subdigital scales between enlarged basal scales and terminal distal pad on digit I of manus and pes**	**3 or 4 small subdigital scales between enlarged basal scales and terminal distal pad on digit I of manus and pes**
SSC	4−6 subdigital scales on claw-bearing segment of digits II−V of manus and pes with distalmost scale markedly larger than the others	**4−6 large and subequal subdigital scales on claw-bearing segment of digits II−V of manus and pes**	**4−6 large and subequal subdigital scales on claw-bearing segment of digits II−V of manus and pes**	**Numerous small scales increasing gradually in size distally**	3−7 subdigital scales on claw-bearing segment of digits II−V of manus and pes with distalmost scale markedly larger than the others
DO	Smooth dorsal scales on body surface smaller than ventrals	Smooth dorsal scales on body surface equal or smaller in size than ventrals	Smooth dorsal scales on body surface smaller than ventrals	**Smooth dorsal scales on body surface equal in size than ventrals**	**Smooth dorsal scales on body surface equal in size than ventrals**
VE	Smooth unpigmented or poorly pigmented ventral scales on body surface	Smooth unpigmented or poorly pigmented ventral scales on body surface	Smooth unpigmented or poorly pigmented ventral scales on body surface	**Smooth pigmented ventral scales on body surface**	**Smooth pigmented ventral scales on body surface**
SC	Smooth pigmented subcaudal scales	Smooth pigmented subcaudal scales	**Smooth unpigmented subcaudal scales**	Smooth pigmented subcaudal scales	Smooth pigmented subcaudal scales
ME	Triangle-shaped mental scale	Triangle-shaped or bell-shaped mental scale	**Bell-shaped mental scale**	Triangle-shaped mental scale	Triangle-shaped mental scale
1PM	Large first large postmental scales in contact for more than 50% of their length	Large first postmental scales in contact for more than 50% of their length	Large first postmental scales in contact for more than 50% of their length	**Large first postmental scales in contact for 50% or less of their length**	Large first postmental scales in contact for more than 50% of their length

### ﻿Molecular differentiation

Phylogenetic analyses were performed on a 16S alignment of 530 bp length and included 66 sequences, 12 of which were newly generated from samples of *P.tsaranoro* sp. nov. and 14 were from newly collected samples of *P.felicitae* (Suppl. material 1). JMODELTEST analysis suggested “GTR+G” as the best model of sequence evolution under all information criteria (Akaike Information Criterion, corrected Akaike Information Criterion, and Bayesian Information Criterion). All species of the genus *Paragehyra* were recovered as monophyletic with the highest statistical support (Posterior Probability (PP) = 1.00), including the new species *P.tsaranoro* sp. nov. (Fig. 4B). *Paragehyrafelicitae* and *P.petiti* formed a strongly supported clade (PP = 1.00), and *P.tsaranoro* sp. nov. was recovered as sister to this (PP = 1.00). *Paragehyraaustini* and *P.gabriellae* formed another strongly supported clade (PP = 1.00), whose relationship with the clade composed of *P.felicitae*, *P.petiti*, and *P.tsaranoro* sp. nov. was not supported (PP = 0.74), which means that the monophyly of the genus *Paragehyra* did not receive strong statistical support with this dataset (Fig. 4B).

The 16S uncorrected *p*-distances showed high inter-specific genetic distances within the genus, and *P.tsaranoro* sp. nov. resulted as clearly differentiated from any other species (Table 3). *Paragehyratsaranoro* sp. nov. showed the lowest 16S distance with *P.felicitae* (6.49%). The largest 16S uncorrected *p*-distances for *P.tsaranoro* sp. nov. was observed between this lineage and *P.gabriellae* (12.93%). Similar to the other congeneric species, *P.tsaranoro* sp. nov. showed low within-species genetic distances (0.36%). This value was close to the levels observed in *P.gabriellae* (0.34%) and slightly lower than the one observed in *P.felicitae* (0.48%) (Table 3). Although based on a much lower number of analysed individuals and lacking sequences of *P.gabriellae*, the uncorrected *p*-distances computed at the COI gene fragment (based on an alignment of 664 bp length and 12 individuals) are concordant with the pattern observed at the 16S marker (Table 3). *Paragehyratsaranoro* sp. nov. showed the lowest inter-specific distance with *P.felicitae* (15.67%) and the highest distance with *P.austini* (20.78%). Similar to the pattern observed at the 16S marker, *P.tsaranoro* sp. nov. had relatively low levels of within-species uncorrected *p*-distances at the COI fragment (1.28%), almost half of the values observed for *P.felicitae* (Table 3).

We generated eleven new sequences of the nuclear markers BDNF and POMC from *P.tsaranoro* sp. nov. and *P.felicitae* (Suppl. material 1). The reconstructed networks (Fig. 4A) showed consistency with phylogenetic analyses in suggesting a closer relationship between *P.felicitae*, *P.petiti*, and *P.tsaranoro* sp. nov. relative to *P.austini* and *P.gabriellae*. We recovered a single haplotype for *P.tsaranoro* sp. nov. in both nuclear markers. The BDNF haplotype was shared with *P.felicitae* and *P.petiti* and two mutational steps distant from *P.austini* and *P.gabriellae*. At the POMC, three mutations separated *P.tsaranoro* sp. nov. haplotype from one of the two haplotypes recovered in *P.felicitae*.

#### ﻿ Paragehyra tsaranoro sp. nov.

Taxon classificationAnimaliaSquamataGekkonidae

7289D03F-015E-5A86-B55E-B513AC249CA1

https://zoobank.org/A79CE326-4EC1-431D-A3D3-BE728E55540E

Figs 3 , 5 , 6 , Tables 1 , 4 , Suppl. material 2

##### Note.

The species has been reported as Paragehyrasp. aff.felicitae “Tsaranoro” CCS in Belluardo et al. (2021a).

##### Type material.

***Holotype*.** Madagascar • 1 adult ♂; south-eastern Madagascar, Haute Matsiatra Region, Fianarantsoa province, ca 32 km south of the town of Ambalavao, Tsaranoro, Forêt Sacrée; 22°04'57.65"S, 46°46'33.56"E; 927 m a.s.l.; 6 Dec. 2018; F. Belluardo, J. Lobón-Rovira, M. Rasoazanany leg.; boulder in semi-deciduous forest close to the forest edge; GenBank: MZ285407; ZSM 11/2023: ACZCV765. Figs 3A, 5. ***Paratypes*.** Madagascar • 1 adult ♀; south-eastern Madagascar, Haute Matsiatra Region, Fianarantsoa province, western slopes of Andringitra National Park, Iantaranomby; 22°07'42.60"S, 46°50'52.90"E; 1,610 m a.s.l.; 19 Nov. 2018; F. Belluardo, J. Lobón-Rovira, G. M. Rosa leg.; boulder in open environment with scattered palm trees; GenBank: MZ285406, PV389999; ZSM 10/2023: ACZCV600. Fig. 3. Madagascar • 1 juv.; south-eastern Madagascar, Haute Matsiatra Region, Fianarantsoa province, Ambatomainty, Tsiomba; 22°00'10.37"S, 46°47'16.08"E; 960 m a.s.l.; 8 Dec. 2018; F. Belluardo, J. Lobón-Rovira, M. Rasoazanany leg.; large boulders in open environment surrounded by scattered trees; GenBank: MZ285516, PV383222; ZSM 12/2023: ACZCV809. Madagascar • 1 adult ♂; same data as preceding; 22°00'09.40"S, 46°47'16.22"E; 947 m a.s.l.; same data as preceding; same data as preceding; same data as preceding; GenBank: MZ285517, PV383221, PV389997; UADBA uncatalogued: ACZCV810. Madagascar • 1 unsexed adult; south-eastern Madagascar, Haute Matsiatra Region, Fianarantsoa province, Tsaranoro Forêt Sacrée; 22°04'51.71"S, 46°46'36.88"E; 909 m a.s.l.; 6 Dec. 2018; F. Belluardo, J. Lobón-Rovira, M. Rasoazanany leg.; large boulders within semi-deciduous forest; GenBank: MZ285518, PV383219, PV389998; UADBA uncatalogued: ACZCV771. Madagascar • 1 unsexed adult; same data as preceding; 22°05'05.03"S, 46°46'30.54"E; 933 m a.s.l.; same data as preceding; same data as preceding; same data as preceding; GenBank: MZ285519, PV383220, PV390005; UADBA uncatalogued: ACZCV770. Madagascar • 1 unsexed adult; same data as preceding; 22°05'07.58"S, 46°46'30.32"E; 983 m a.s.l.; same data as preceding; same data as preceding; same data as preceding; GenBank: MZ285520, MZ285408; UADBA uncatalogued: ACZCV779.

##### Type locality.

Tsaranoro Forêt Sacrée (south-eastern Madagascar, Haute Matsiatra Region, Fianarantsoa province, ca 32 km south of the town of Ambalavao), 22°04'57.65"S, 46°46'33.56"E, 927 m a.s.l. A semi-deciduous forest fragment of ca 46 ha within Tsaranoro Valley Forest reserve (Figs 1, 2).

##### Diagnosis.

The species is assigned to the genus *Paragehyra* based on genetic distances at the COI and 16S markers, the presence of two diagnostic morphological derived characters of the genus relative to the other gekkonids: the asymmetrical relationship of the claw and toe-pad on digit I and the uniscansorial distal pad on digit I separated from enlarged basal scales by a series of smaller scales (Nussbaum and Raxworthy 1994; Crottini et al. 2015). The inter-specific diagnosis is detailed in the following lines (Fig. 6; Tables 1, 4).

*Paragehyratsaranoro* sp. nov. has 12 distinct longitudinal rows of enlarged tubercles on the body dorsolateral surface (character BT), enlarged tubercles on the entire dorsal surface of hind limbs and on distal dorsal portions of forelimbs (character TDL), enlarged tubercles organised in transversal rows that encircle tail dorsolateral surface (character TT), 6−8 enlarged infralabial scales gradually decreasing in size in posterior direction (character I), lateral chin scales extending forward along each side, excluded from contact with first line of infralabials and first postmentals, in contact with second line of infralabials (character C), 3−6 ventral scales on the distal (tibial) segments of hind limbs enlarged into plates especially in the most distal part (character VET), five or six large scales on preaxial ventral portion of pes between end of tibia and base of digit I (character SPP), two or three small subdigital scales between enlarged basal scales and terminal distal pad on digit I of manus and pes (character SS), 4−6 subdigital scales on claw-bearing segment of digits II−V of manus and pes with the distalmost scale markedly larger than the others (character SSC), smooth dorsal scales on body surface smaller than ventral scales (character DO), smooth unpigmented or poorly pigmented ventral scales on body surface (character VE), smooth and pigmented subcaudal scales (character SC), triangle-shaped mental scale (character ME), large first postmental scales in contact for more than the 50% of their length (character 1PM).

Among species of the genus *Paragehyra*, *P.felicitae* is the most similar to *P.tsaranoro* sp. nov., from which it differs in the following four morphological characters: TDL, enlarged tubercles present on the entire dorsal surface of hind limbs and only on distal dorsal segments of forelimbs (vs enlarged tubercles present on the entire dorsal surface of both hind limbs and forelimbs); C, lateral chin scales extend forward along each side, excluded from contact with first line of infralabials and first postmentals, in contact with second line of infralabials (vs lateral chin scales extend forward along each side, excluded from contact with first line of infralabials and first postmentals, in contact with second line of infralabials or lateral chin scales extend forward along each side, excluded from contact with first infralabials, in contact with first postmentals and second infralabials); SPP, five or six large scales on preaxial ventral portion of pes between end of tibia and base of digit I (vs generally 6−9 large scales on preaxial-ventral portion of pes between end of tibia and base of digit I); SSC, 4−6 subdigital scales on claw-bearing segment of digits II−V of manus and pes with the distalmost scale markedly larger than the others (vs 4−6 large and subequal subdigital scales on claw-bearing segment of digits II−V of manus and pes).

*Paragehyratsaranoro* sp. nov. differs from *P.petiti* in the following five characters: BT, 12 (vs 10) distinct longitudinal rows of enlarged tubercles on dorsolateral body surface; I, 6−8 enlarged infralabials that diminish gradually in size posteriorly (vs first 4−6 infralabials much larger than remainders); SSC, enlarged tubercles present on the entire dorsal surface of hind limbs and only on distal dorsal segments of forelimbs (vs 4−6 large and subequal subdigital scales on claw-bearing segment of digits II−V of manus and pes); SC, smooth and pigmented subcaudal scales (vs smooth and unpigmented subcaudal scales); ME, triangle-shaped mental scale (vs bell-shaped mental scale).

*Paragehyratsaranoro* sp. nov. differs from *P.austini* for the following eight characters: BT, 12 distinct longitudinal rows of enlarged tubercles on dorsolateral body surface (vs absence of any tubercle on dorsolateral body surface); TDL, enlarged tubercles present on the entire dorsal surface of hind limbs and only on distal dorsal segments of forelimbs (vs absence of enlarged tubercles on limbs dorsal surface); TT, transverse rows of enlarged tubercles that encircle tail dorsolateral surface (vs absence of tubercles on tail dorsolateral surface); VET, 3−6 ventral scales on the distal (tibial) segments of hind limbs enlarged into plates especially in the most distal part (vs ventral scales on distal (tibial) segment of hind limb slightly larger compared with scales on the proximal segments of hind limbs); SPP, five or six large scales on preaxial ventral portion of pes between end of tibia and base of digit I (vs generally 7 small scales on preaxial-ventral portion of pes between end of tibia and base of digit I); SS, two or three small subdigital scales between enlarged basal scales and terminal distal pad on digit I of manus and pes (vs 3 or 4 small subdigital scales between enlarged basal scales and terminal distal pad on digit I of manus and pes); DO, smooth dorsal scales on body surface smaller than ventral scales (vs smooth dorsal scales on body surface equal in size than ventrals); VE, smooth unpigmented or poorly pigmented ventral scales on body surface (vs smooth pigmented ventral scales on body surface).

*Paragehyratsaranoro* sp. nov. differs from *P.gabriellae* in the following 11 characters: BT, 12 distinct longitudinal rows of enlarged tubercles on dorsolateral body surface (vs presence of small tubercles not arranged in distinct rows); TDL, enlarged tubercles present on the entire dorsal surface of hind limbs and only on distal dorsal segments of forelimbs (vs absence of enlarged tubercles on limbs dorsal surface); TT, transverse rows of enlarged tubercles that encircle tail dorsolateral surface (vs presence of enlarged tubercles on tail not organised in transverse rows); C, lateral chin scales extend forward along each side, excluded from contact with first line of infralabials and first postmentals, in contact with second line of infralabials (vs lateral chin scales extend forward along each side in contact with first and second infralabials and with first postmentals or lateral chin scales excluded from contact with first infralabials, in contact with first postmentals and second infralabials); VET, 3−6 ventral scales on the distal (tibial) segments of hind limbs enlarged into plates especially in the most distal part (vs ventral scales on distal (tibial) segment of hind limb of normal size compared with scales on the proximal segments of hind limbs); SPP, five or six large scales on preaxial ventral portion of pes between end of tibia and base of digit I (vs 7−9 small scales along preaxial-ventral border of pes between end of tibia and base of digit I); SS, two or three small subdigital scales between enlarged basal scales and terminal distal pad on digit I of manus and pes (vs 7−10 small subdigital scales between enlarged basal scales and terminal distal pad on digit I of manus and pes); SSC, 4−6 subdigital scales on claw-bearing segment of digits II−V of manus and pes with the distalmost scale markedly larger than the others (vs numerous small scales increasing gradually in size distally); DO, smooth dorsal scales on body surface smaller than ventral scales (vs smooth dorsal scales on body surface equal in size than ventrals); VE, smooth unpigmented or poorly pigmented ventral scales on body surface (vs smooth pigmented ventral scales on body surface); 1PM, large first postmental scales in contact for more than the 50% of their length (vs large first postmental scales in contact for 50% or less of their length).

##### Description of the holotype.

Figs 3A, 5, 6; Table 1; Suppl. material 2. The holotype is an adult male in well-preserved condition with intact and original tail. Hemipenes are not everted. Digit III of the right pes was clipped and stored in 96% ethanol as tissue sample for molecular analyses.

The specimen has flattened body and head. Head width is slightly lower than body width (11.95 mm vs 13.65 mm). The head snout is rounded, with HL and HW that are 0.32 and 0.21× SVL, respectively. Head is 1.52 longer than wider (HL/HW), 3.3× longer than deeper (HL/HD), and 2.17 larger than deeper (HW/HD). Eye with vertical pupil and ED that is 0.29× relative to SnL and 0.66× the IOD. Ear openings are elliptical in vertical direction with a diameter on the horizontal axis (EO) that is 0.33× the ED and 5.05 mm distant from the eye (ETD). Forelimb and hind limb lengths are 0.32 and 0.48× SVL, respectively. Forelimbs and hind limbs lengths are 0.72 and 1.08× the AGD, respectively. Forelimb when extended forward reaches nostril, when extended posteriorly reaches three quarters of distance to groin, hind limb reaches anterior axilla. Tail length is 1.24× SVL. The tail is subcylindrical and dorsoventrally flattened at its base with a pointed and narrow tip.

A concave groove is present between nasal scales, which are not in direct contact, separated by one IN. Nostrils in contact with rostral, nasals, and four postnasals, not in contact with first supralabials. Quadrangular rostral scale less wide than mental scale with an incomplete, dorsal vertical groove extending downward approximately one-half the distance from dorsal edge to lip. Nine enlarged supralabial scales (mostly rectangular) are present on both right and left sides (SL), and eight and seven infralabial scales (I) are on the right and left sides, respectively. Infralabials gradually decrease in size posteriorly. The first six supralabials are squared and have equal size, while the last three scales are much smaller and elongated in posterior direction. Triangle-shaped ME laterally in contact with the first line of infralabials. Posterior to the mental scale are two large 1PM with irregular pentagonal shapes and in reciprocal contact for more than 50% of their length. The first postmentals are bordered posteriorly by a line of six smaller polygonal scales (2PM, which are clearly distinguishable from the much smaller and mostly granular chin scales that are posterior to these) and in contact with the first line of I. Scalation of the contact between chin scales with postmentals and infralabials (C) is variable between left and right sides. On the left side, chin scales extending laterally are excluded from contact with the first line of infralabials and first postmentals and are in contact with the second line of elongated infralabials (defined as a distinguishable line of scales that are larger than chin scales and parallel to the first infralabials). On the right side, chin scales extending laterally are in contact with a third line of elongated infralabials but excluded from contact with the first and second lines of infralabials and first postmentals. Throat scales small, circular, and largely juxtaposed. Scales just below posterior infralabials enlarged. Throat covered by small granular scales.

Scales on the dorsal body surface are mostly small granular with smooth surface, smaller than ventral scales (approximately half dimension on the horizontal axis). Dorsal enlarged body tubercles (~ three times bigger than scales) with rounded or subconical shape that are arranged into 12 distinct parallel longitudinal rows (BT), equally divided between the two body sides. Dorsal scales on limbs are mostly small with granular shape and smooth surface. Body tubercles extend on the entire surface of hind limbs and only on the distal portions of forelimbs (TDL). Hind limbs tubercles are larger than tubercles on forelimbs and larger than the dorsal tubercles organized in the 12 parallel longitudinal rows. A few scattered tubercles are also found on the posterior-lateral portion of the head and the neck. Dorsal scales on the tail are small and granular with smooth surface and of the same size as the dorsal scales on body. Eight dark whorls with one or two transverse rows (ring) of enlarged tubercles of each of the first five dark whorl are alternated until the tail tip with seven paler whorls with two transverse rows (rings) of enlarged tubercles, one at the anterior and one at the posterior border of each of the first four pale whorl. Enlarged tubercles on tail are larger than the dorsal tubercles organised in the 12 parallel longitudinal and the enlarged tubercles present of limbs. Whorl 1 and 2 have 12 enlarged tubercles, whorls 3 and 4 have six enlarged tubercles, whorls 5 and 6 have four enlarged tubercles, whorl 7 has three enlarged tubercles, whorl 8 has two enlarged tubercles, whorl 9 has a single enlarged tubercle. The two distalmost pale whorls are lighter than the previous ones. Dark whorls size increases in posterior direction. Pale whorl size increases in posterior direction until the fourth pale whorl, with the remaining three pale whorls gradually decreasing towards the tail tip. Transverse rows of enlarged tubercles with rounded or subconical shape encircle the tail dorsolateral surface (TT). Tubercle size gradually decreasing in posterior direction.

Scales on the body ventral surface are mostly regular rhombus-shaped and juxtaposed with smooth surface (VE). They are unpigmented or poorly pigmented (pigments are visible only under stereo microscope). Slightly imbricate, cycloid scales begin behind throat and cover chest and belly. Ventral surface of forelimbs covered with granular scales on proximal segment and imbricate cycloid scales on distal segment. Hind limbs have mostly large polygonal and slightly imbricate scales with smooth surface and unpigmented to the naked eye, as described above for the ventral body surface. Some pale scale pigmentation is only visible on belly. Ventral surface of pelvis and thigh (proximal portion of hind limbs) covered with imbricate cycloid scales. Scales on the hind limbs distal (tibial) portion are larger than on the proximal portion. They gradually increase in size towards pes, and the three distalmost form large plates (VET) as large as half the limb width. Subcaudal scales are enlarged transversely, smooth, and pigmented, especially in their anterior and posterior borders (SC). In the distalmost part of the tail, they tend to be pigmented also in their central portions and follow the alternated dark and pale colour pattern of the dorsal whorls described above. In the proximal portion of the tail, there are three central longitudinal rows of large imbricate and mostly cycloid scales and, starting from the section corresponding to the second dark dorsal whorl and in posterior direction, they form a unique longitudinal row of imbricate undivided plates almost as large as the tail width. These central longitudinal rows are laterally bordered on both sides by 1 to 3 longitudinal rows of smaller imbricate cycloid scales. Scales immediately adjacent to cloacal opening much smaller than surrounding scales. Precloacal or femoral pores are absent.

Preaxial border of palm and digit I of manus covered with large scales. The pes palm is covered with mostly granular scales in their central part and relatively large cycloid imbricate scales on the lateral portions. In particular, on the pes preaxial ventral portion between the end of the tibia and the base of digit I there is a longitudinal row of five (right) and six (left) large imbricate cycloid scales with smooth surface on pes (SPP). Subdigital scalation of digit I of manus and pes organised into a basal longitudinal row of relatively large imbricate scales on the proximal portion, followed by two intermediate parallel rows of smaller juxtaposed scales (composed of three scales on left manus and pes; SS), and a distal enlarged rectangular pad with an extremely small claw (only visible with a stereo microscope) on top of it. Mostly imbricate cycloid scales on basal portions of digits II−V of manus and pes followed by rows of undivided and transversely enlarged pads covering the distal two-thirds of digits. Lamellae cover the most distal pads of digits II−V with the following numbers on the left side: 6, 7, 7, 7 pads (manus), 8, 8, 7, 7 pads (pes) (SLMP). Claw-bearing segments on top of the subdigital lamellae composed of relatively large imbricate rectangular scales organised into longitudinal rows with some degree of overlap between them. In the central row, the distalmost scale is markedly larger than the others and composed of the following scale numbers on left digits II−V: 5,5,5,5 (manus) and 5,6,5,5 (pes) (SSC). Comparative finger and toe length in manus is 1<2<5<3<4, in pes 1<2<3<4<5.

##### Colouration.

Colouration after six years in ethanol is slightly paler but with an identical pattern to that at the time of collection (Figs 3A, 5, 6). Dorsolateral surface of the head, body, limbs, and tail with a pale grey ground colour and dark brownish transversal bands and blotches. Head with large posterior blotches and scattered dark brownish dots and linear marks until the mouth tip. A dark brownish line connects ear openings to the eyes posteriorly and reaches half of the distance between the eye anterior parts and nostrils, on both sides. Brownish longitudinal vertebrate line on the dorsal body surface. Five transversal dorsolateral dark brownish blotches on the dorsal body surface with a paler greyish spot in their centre. Hind limbs with transverse bands that are more defined in their distal (tibial) rather than proximal portions, where they can be more similar to blotches. Forelimbs with less defined transverse bands with interspersed brownish blotches. Dorsal surface of digits with two pale grey and two dark brownish bands alternated from the base until the tip; in life colouration, the second pale band towards the distal portion is white. In the preserved specimen the tail has alternated dorsolateral pale grey and dark brownish transverse bands. The ventral surface of the head, body, limbs, and tail is uniformly pale brownish tending to whitish, for the presence of a sparse dotted dark pigmentation visible only under stereo microscope (character VE; Fig. 6). Ventral (subdigital) surface of digits is slightly darker than ground colour, and lamellae are white. Subcaudal scales borders are slightly darker than the ground colour. The distal part of the tail (ca one-third of the entire length) has alternated dark brownish and ground colour bands, and the tip is black. Supralabials and infralabials with alternated whitish and dark brownish vertical bands, with more intense dark colouration on supralabials (Fig. 6).

##### Variation.

Variation in morphological characters of ZSM paratypes is reported in Table 1 and Suppl. material 2 (see also Fig. 3). After six years in ethanol, colouration of the paratypes ZSM 10/2023 (ACZCV600) and ZSM 12/2023 (ACZCV809) is slightly paler to that in life and overall similar to the holotype. Paratype ZSM 10/2023 (ACZCV600) differs from the holotype in the following characters: nasal scales are in contact, 11 enlarged left supralabials (SL), six left I, eight 2PM, scalation of chin scales identical between right and left sides with lateral chin scales extending forward excluded from contact with first line of infralabials and first postmentals but in contact with second line of infralabials (C), six plates on ventral side of distal (tibial) segments of hind limbs (VET), and two scales compose the intermediate longitudinal rows between enlarged basal scales and distal pad of digit I of pes (SS). Refer to Table 1 and Suppl. material 2 for the variation in morphological measurements and in SSC and SLMP . The head dorsal surface is slightly darker than in the holotype, with a characteristic pale W-shaped blotch posterior to the eyes and two central pale spots anterior to the eyes. The specimen does not have the tail, and the third digit of the right pes was clipped as tissue sample. Paratype ZSM 12/2023 (ACZCV809), an unsexed juvenile, differs from the holotype in the following characters: nasal scales are in contact (IN), 10 enlarged left SL, eight right I, modified bell-shaped ME, eight second 2PM, scalation of chin scales identical between right and left sides with lateral chin scales extending forward excluded from contact with first line of infralabials and first postmentals but in contact with second line of infralabials (C), four plates on ventral side of distal (tibial) segments of hind limbs (VET), six scales on preaxial-ventral portion of right pes between end of tibia and base of digit I (SPP), and two scales make the intermediate longitudinal rows between enlarged basal scales and distal pad of digit I of manus (SS). Refer to Table 1 and Suppl. material 2 for the variation in morphological measurements and in the number of subdigital scales and subdigital lamellae on claw-bearing segments of digits II−V (SSC and SLMP, respectively). Morphological variation and measurements are not available for the paratypes hosted in the UADBA collection: UADBA uncatalogued (ACZCV810), UADBA uncatalogued (ACZCV771), UADBA uncatalogued (ACZCV770), and UADBA uncatalogued (ACZCV779).

##### Etymology.

The specific epithet derives from the type locality Tsaranoro. The name is used as an invariable noun in apposition to the generic name.

##### Distribution.

*Paragehyratsaranoro* sp. nov. is currently known from south-eastern Madagascar, restricted to three localities within the western part of the region surrounding the Andringitra Massif (Haute Matsiatra administrative region, Fianarantsoa province): Tsaranoro, Ambatomainty, and Iantaranomby (Suppl. material 1; Figs 1, 2). Iantaranomby is on the western slope of the Andringitra Massif, while Tsaranoro and Ambatomainty forests are in the plateau located west to the Massif, laying at the foot of two granitic domes rising a few hundred meters relative to ground elevation. The species has been sampled within an elevation range between 897 m (Ambatomainty) and 1,610 m a.s.l. (Iantaranomby).

##### Habitat and behaviour.

The holotype ZSM 11/2023 (ACZCV765) was found at night (~ 8 p.m.) in the semi-deciduous forest fragment of Tsaranoro under clear weather. The animal was resting on a boulder near the forest edge. Nearby, we sampled an individual of *Paroedurarennerae* Miralles, Bruy, Crottini, Rakotoarison, Ratsoavina, Scherz, Schmidt, Köhler, Glaw & Vences, 2021 (Belluardo et al. 2021a). The remaining individuals of *P.tsaranoro* sp. nov. from Tsaranoro forest were found on boulders. Paratype UADBA uncatalogued (ACZCV771) was collected outside the forest patch along a small stream amidst arboreal riverine vegetation. The other two paratypes, UADBA uncatalogued (ACZCV770 and ACZCV779), were found within the forest interior, along with additional individuals not collected as vouchers (Suppl. material 1). All Tsaranoro animals were found at night, except for ACZC10973, collected in the morning (~ 9 a.m.). Paratype ZSM 10/2023 (ACZCV600) was the only individual collected within Andringitra NP (Iantaranomby, ca 400 m from the park limit; Fig. 2C). This animal was found at night in clear weather under a large boulder on a sloping trail surrounded by scattered palm trees. The three individuals from Ambatomainty Tsiomba (including the paratypes UADBA uncatalogued (ACZCV810) and ZSM 12/2023 (ACZCV809)) were all found outside the small forest patch (Fig. 2A). These animals were sampled at night in clear weather on large boulders in open areas near the forest fragment surrounded by scattered trees.

Overall, *P.tsaranoro* sp. nov. seems to be associated with semi-deciduous forest. The species is found both within the forest interior and along its edges, as well as outside the forest or along water streams, but always on boulders associated with arboreal vegetation. The isolated forest fragments in the study area are characterised by numerous boulders, many of which are ancient Betsileo tombs (Gould and Andrianomena 2015). This cultural significance has led to these areas being referred to as “Forêts sacrées”, i.e., “sacred forests”. The boulders vary in size, sometimes forming large agglomerates, with some reaching considerable dimensions. The higher number of individuals found in the interior of Tsaranoro forest compared to other sites suggests that relatively larger and more mature forests may support higher population densities of this species.

##### Proposed conservation status.

The extent of occurrence (EOO) and area of occupancy (AOO) total 38.8 km^2^ and 16 km^2^, respectively (computed with GeoCAT (Bachman et al. 2011) using squared grid cells of 2 × 2 km for AOO). Following IUCN Red List guidelines (IUCN Standards and Petitions Committee 2024), we propose to consider *P.tsaranoro* sp. nov. as Critically Endangered (CR) under criterium B1ab(iii). The proposed evaluation of the species conservation status is justified by the narrow distribution (EOO < 100 km^2^), its occurrence at three severely isolated localities, and the continuing decline in the extent and quality of its habitat due to specific threats.

The habitat of this species is severely fragmented, with the three known localities isolated by unsuitable landscapes of villages, rice fields, and pastures. In two of these localities, forests areas and arboreal vegetation are heavily degraded. The Ambatomainty forest is extremely small (ca 2 ha) with almost no canopy cover. In Iantaranomby (Andringitra NP), the single specimen encountered was found in an area with only a few scattered palm trees. Unlike Ambatomainty and Iantaranomby, Tsaranoro forest provides particularly favourable conditions for the species, featuring well-structured vegetation and abundant boulders. Tsaranoro is also the largest forest fragment in the region (46 ha; Gould and Andrianomena 2015; Gould and Gabriel 2015), likely contributing to the higher abundance of the species observed there.

Iantaranomby is the only locality with formal legal protection, located within Andringitra NP borders. No legal protection or local management is known for Ambatomainty. Tsaranoro forest is managed by the local community Association Tantely since 2002. Although in the past the forest has been subject to selective logging (Gabriel et al. 2018), reforestation projects consisting in the establishment of tree nurseries have been recently funded (Gould and Andrianomena 2015). However, and apart from Tsaranoro, deforestation in the region has been particularly intense and seems to be a recent and still ongoing process. Most deforestation occurred in the last 60 years and until 20 years ago there were still remnant parts of continuous forests (Gould and Andrianomena 2015; Gould and Gabriel 2015). The process of deforestation and forest degradation in the region is continuing through direct tree logging (normally used as firewood) and fires that are normally used to clean areas for cattle grazing and that sometimes go out of control and affect the remaining forest fragments (Crottini et al. 2015; Gould and Andrianomena 2015; Gould and Gabriel 2015). We could directly observe these activities in some of these fragments, even within the legally protected area of Andringitra NP. Ongoing deforestation, forest degradation, and fragmentation seem to be the main threats to the conservation of the small forest fragments where *P.tsaranoro* sp. nov. lives and their persistence is expected to have an impact on this newly described gecko species.

##### Updated distribution and proposed conservation status of *P.felicitae*.

When *P.felicitae* was formally described, the species was only known from its type locality within Anja reserve and a few boulders located ca 1 km away, on the opposite side of the national road RN7 relative to the reserve (Fig. 2F; Suppl. material 1; Crottini et al. 2015). Due to the limited knowledge of the species distribution within the areas surrounding Anja and following IUCN Red List guidelines, Crottini et al. (2015) proposed to consider the species as Data Deficient. During the herpetological survey conducted by Belluardo et al. (2021a) in several localities of the Andringitra region, including several areas surrounding Anja, the species was confirmed within Anja reserve and a few individuals were found in the forest of Sakaviro reserve (Fig. 2E; Suppl. material 1), located ca 8 km north of Anja (Fig. 1). The semi-deciduous forest in Sakaviro is smaller than in Anja (14 vs 36 ha, Gould and Andrianomena 2015), and host similar environmental characteristics, being located at the base of a small granitic peak and with the presence of several large granitic boulders. Species elevation ranges between 950 m (Anja) and 1,089 m a.s.l. (Sakaviro).

With the improved knowledge on the species distribution in the region surrounding the type locality, it is now possible to propose a new conservation status for *P.felicitae*. The updated EOO and AOO total 5 km^2^ and 16 km^2^, respectively. The EOO has been adjusted to 16 km^2^ as it cannot be smaller than AOO (IUCN Standards and Petitions Committee 2024). Although smaller grid cells might produce lower and more realistic AOO extents, we followed IUCN Red List guidelines in using 2 × 2 km cells. We propose to evaluate this species as CR under criterium B1ab(iii) based on the narrow distribution (EOO < 100 km^2^), the occurrence at three isolated threat-defined locations (Anja reserve, the few boulders located at ca 1 km away from Anja and Sakaviro reserve), and the continuing decline in the extent and quality of its habitat due to persistent deforestation and forest degradation in the surrounding areas.

Similar to *P.tsaranoro* sp. nov., *P.felicitae* seems to be associated with granitic boulders within or close to semi-deciduous forest (Crottini et al. 2015). Despite their limited extensions, both Anja and Sakaviro forests seem to host suitable habitats and the species was found to be relatively abundant in both localities. Anja and Sakaviro forests are managed by local community associations: Anja Miray (since 2000) and Sakaviro Miray (since 2012), respectively (Gould and Andrianomena 2015). Anja forest has been subject to selective logging and introduction of non-native trees before the community-managed reserve became established (Gabriel et al. 2018). Some human disturbance at the forest edge (i.e., tree logging and cattle grazing) has been observed (Crottini et al. 2015), suggesting that some level of forest use for livelihood resource remains, as expected in community-managed forests (Raik 2007; Nopper et al. 2017). The combination between severe habitat fragmentation and continued widespread deforestation within the region surrounding Anja and Sakaviro and some level of human disturbance in these forests (Crottini et al. 2015; Gould and Andrianomena 2015; Gould and Gabriel 2015) represent the main threats to *P.felicitae* and might seriously impact the viability of local populations causing the long-term decline of the species.

## ﻿Discussion

The genus *Paragehyra* has been poorly studied compared to other species-rich genera of Malagasy geckos (e.g., Miralles et al. 2021; Vences et al. 2022b; Piccoli et al. 2023; see Bauer et al. 2022; Gehring and Ratsoavina 2022; Gehring et al. 2022), and the phylogenetic relationships of this genus in relation to other gekkonids from Madagascar and from the rest of the world remain substantially unresolved (Gamble et al. 2015; Zheng and Wiens 2016; Bauer et al. 2022). After two taxonomic revisions and the description of new species, four nominal species were included in the genus *Paragehyra* until now (Nussbaum and Raxworthy 1994; Crottini et al. 2015; Uetz et al. 2025). In this study, we described *P.tsaranoro* sp. nov., a new microendemic species from the western part of the Andringitra Massif region. We justified the description of *P.tsaranoro* sp. nov. following an integrative taxonomic approach combining multiple lines of evidence (Padial et al. 2010). At the genetic level, the large uncorrected *p*-distances of mitochondrial markers (16S and COI) and the absence of haplotype sharing at the POMC nuclear marker differentiated *P.tsaranoro* sp. nov. from congeneric species (Table 3; Fig. 4). At the morphological level, the species is distinguishable from all other congeneric species by a combination of 14 morphological characters (Tables 1, 4). We also provided new genetic and morphological data of *P.felicitae* from newly collected tissue samples and voucher specimens (Tables 1, 3, Suppl. materials 1, 2, Fig. 4; Belluardo et al. 2021a). These new data enabled us to update the state definitions of the characters C, VET, SPP, DO and ME relative to the species description (Table 4; Crottini et al. 2015).

The record of *P.tsaranoro* sp. nov. from Iantaranomby extends by ca 600 m the upper elevational limit of the genus, which now ranges from 15 m a.s.l. (*P.gabriellae* from Manantantely; Suppl. material 1) to 1,610 m a.s.l., suggesting that despite the limited species richness, *Paragehyra* geckos are adapted to a relatively wide range of temperatures and climatic conditions. Similarly, the records from Sakaviro extend the upper limit of the elevational range of *P.felicitae* from 1,000 m a.s.l. (so far considered the highest record of the genus; Crottini et al. 2015) to 1,089 m a.s.l. (Suppl. material 1).

In our phylogeny, the inter-specific relationships within the genus *Paragehyra* (Fig. 4B) agree with the previous phylogenetic hypothesis inferred by Crottini et al. (2015) based on a larger multi-locus dataset of mitochondrial and nuclear markers. However, differently from this study, in our analysis the sister relationship between *P.austini* and *P.gabriellae* was statistically supported, while we could not retrieve support for the monophyly of the genus. Interestingly, the reciprocal closest morphological similarity between *P.felicitae* and *P.tsaranoro* sp. nov. (Table 4) is not reflected at the genetic and phylogenetic level (Table 3; Fig. 4), where *P.felicitae* shows the lowest distances and closest phylogenetic position to *P.petiti* (Table 3; Fig. 4). While all *Paragehyra* species have similar rupicolous microhabitat preferences (Glaw and Vences 2007; Crottini et al. 2015), the two main sub-clades have clear distinct macroclimatic and macrohabitat preferences: *P.austini* and *P.gabriellae* inhabit humid forests, while the subclade composed of *P.felicitae*, *P.petiti*, and *P.tsaranoro* sp. nov. lives in more arid habitats. When Nussbaum and Raxworthy (1994) described *P.gabriellae* and re-described *P.petiti*, they suggested that, given the more plesiomorphic condition of *P.gabriellae* (based on morphological data), it was likely that the common ancestor of the genus inhabited humid forests, as does *P.gabriellae*. However, after the descriptions of *P.austini*, *P.felicitae* (Crottini et al. 2015), and *P.tsaranoro* sp. nov., we can propose a different hypothesis. Assuming that the current species macrohabitat preferences and distributions are indicative of the mechanisms responsible for the diversification of the genus, we can hypothesise that their common ancestor was ecologically tolerant and widespread between eastern humid and western and central drier areas of the island and that, over time, populations living in the two distinct macrohabitats diverged into separate species following adaptive speciation in allopatry, and later diverged into the species that are now part of the two main subclades. This mechanism is known as “Ecogeographic constraint” and is among the main processes that have been proposed to explain the diversification of Malagasy vertebrate fauna (Yoder and Heckman 2006; Vences et al. 2009). Considering that both *P.felicitae* and *P.tsaranoro* sp. nov. inhabit semi-deciduous forests at mid-high altitudes, it is possible that the common ancestor of their subclade (which also includes *P.petiti*) had similar habitat preferences. The diversification in this sub-clade might have occurred in allopatry (as suggested by Crottini et al. 2015), with *P.petiti* specialising in occupying coastal calcareous areas in south-western Madagascar and *P.felicitae* and *P.tsaranoro* sp. nov. that came later into secondary sympatric contact.

*Paragehyrafelicitae* and *P.tsaranoro* sp. nov. live in the same region at ca 20 km of distance. We cannot exclude that future investigations in the same area might uncover new geographic records and even syntopic localities where the two species live in direct contact, although the high levels of forest loss and fragmentation in the region (Gould and Andrianomena 2015; Gould and Gabriel 2015) do not facilitate the identification of contact zones. Despite their limited extensions and isolation, the forest fragments in this area host a remarkable herpetological diversity (Belluardo et al. 2021a). During the recent survey conducted by Belluardo et al. (2021a), six reptile species previously unknown from the region of the Andringitra Massif were found in the same forest fragments as *P.felicitae* and *P.tsaranoro* sp. nov. Among these, probably the records of the chameleon *Furcifernicosiai* Jesu, Mattioli & Schimmenti, 1999 from Tsaranoro and the snake candidate species *Pseudoxyrhopus* sp. Ca2 from Ambatomainty represent the most relevant range extensions (ca 300 km and 230 km, respectively; see Belluardo et al. 2021a, 2021b). Probably the most important aspect supporting the relevant herpetological value of these forest fragments is the presence of several microendemic species that do not occur in any other part of the country: namely *Phelsumagouldi* Crottini, Gehring, Glaw, Harris, Lima & Vences, 2011, *Brookesiabrunoi* Crottini, Miralles, Glaw, Harris, Lima & Vences, 2012, and *P.felicitae* (originally described from Anja; Crottini et al. 2011b, 2012a, 2015), and *Paroeduramanongavato* Piccoli, Belluardo, Lobón-Rovira, Oliveira Alves, Rasoazanany, Andreone, Rosa & Crottini, 2023 from Anja and Tsaranoro (Belluardo et al. 2021a; Miralles et al. 2021; Piccoli et al. 2023). Considering other microendemic species recently described from forest fragments in other parts of the country (e.g., *Calummatarzan* Gehring, Pabijan, Ratsoavina, Köhler, Vences & Glaw, 2010 or *Calummajuliae* Prötzel, Vences, Hawlitschek, Scherz, Ratsoavina & Glaw, 2018; Gehring et al. 2010; Prötzel et al. 2018), this suggests that herpetological research should not overlook regions highly impacted by human activities and mostly lacking legal protection (see also D’Cruze et al. 2009; Vences et al. 2022a). At the same time, the record of *P.tsaranoro* sp. nov. from Andringitra NP (Iantaranomby) demonstrates that even areas that received relatively higher scientific attention still hold the potential to uncover new herpetological diversity (Goodman et al. 2018; Belluardo et al. 2021a), suggesting that the inventory of Malagasy reptiles remains incomplete (Nagy et al. 2012; Antonelli et al. 2022; Glaw et al. 2022).

Considering the restricted distributions of *P.tsaranoro* sp. nov. and *P.felicitae*, their isolated local populations, and persisting threats to their long-term viability posed by the worsening condition in the extent and quality of their habitat, we proposed to list both species as CR (IUCN Standards and Petitions Committee 2024). The largest forest fragments of the region, which are also the largest patches where the two species are known to exist, are community-managed reserves (i.e., Anja, Sakaviro, and Tsaranoro). Despite the possible presence of some level of human disturbance (Crottini et al. 2015), Anja and Tsaranoro represent overall successful examples of local forest management and ecotourism promotion, whose monetary benefits contribute to help local communities (Gould and Andrianomena 2015). Its location on the RN7, one of the main national roads of the country, and the possibility to easily observe a dense population of *Lemurcatta* Linnaeus, 1758, made Anja one of the most visited community-managed reserves of Madagascar (Crottini et al. 2015; Gould and Andrianomena 2015). Tsaranoro is less easily accessible, but it is on the road to the entrance of the western side of Andringitra NP and has become a renowned destination for rock-climbing and para-gliding (Gould and Andrianomena 2015). In other parts of Madagascar, local management of natural resources has not always been as effective as expected and has not necessarily reduced deforestation (see Rasolofoson et al. 2015; Gardner et al. 2020; Long et al. 2021). Although forest fragments managed by local communities can host interesting and unique reptile communities, the impact caused by their use for livelihood resources and limited extensions can alter reptile community composition, with rare forest specialists that might disappear over time (Gardner et al. 2016; Nopper et al. 2017). Forest patches in community-managed reserves cannot have the same conservation value and be alternative to larger and more pristine forests within legally protected areas, such as National Parks, whose main goals are to protect biodiversity (Wright 2010; Nopper et al. 2017). However, they can still play a fundamental role in conserving local herpetofauna, complementary to the national network of protected areas (Jenkins et al. 2014; Nopper et al. 2017). In the case of the region surrounding the Andringitra Massif, the herpetological value of the community-managed reserves (Belluardo et al. 2021a) suggests that these forests can actually complement the protection of Andringitra NP by acting as refugia to local herpetofauna in a landscape that is unsuitable for most of its extension. Therefore, any initiative to favour the persistence and improve the effectiveness of these reserves should be strongly supported.

## ﻿Conclusions

We formally described *P.tsaranoro* sp. nov., a new Malagasy gecko microendemic to the western part of the Andringitra Massif region. We provided multiple independent molecular and morphological lines of evidence to justify its distinctness from all other congeneric species. We also provided new genetic and morphological data of *P.felicitae*. By doing this, we expanded the morphological and molecular datasets of the genus available in Crottini et al. (2015), thus facilitating any future identification of candidate species, the evaluation of their taxonomic status and formal description. However, the knowledge on the genus *Paragehyra* is still very limited. To test specific diversification hypotheses within a robust statistical framework, more fieldwork efforts are needed to collect data on species natural history and improve knowledge on their distribution. Generating additional genetic (and possibly genomic) data might help clarifying the taxonomic identity of the population of *Paragehyra* from Tsingy de Bemaraha (Crottini et al. 2015).

We propose to classify both *P.tsaranoro* sp. nov. and *P.felicitae* in the CR category of the IUCN Red List (IUCN Standards and Petitions Committee 2024), due to their small EOOs, the high levels of forest loss and fragmentation in the region where these species are found and the continuing decline in the extent and quality of their habitat (Gould and Andrianomena 2015; Gould and Gabriel 2015). The fact that both *P.tsaranoro* sp. nov. and *P.felicitae* mostly occur and are apparently more abundant within forests fragments managed by local communities suggest that this form of local management of natural resources can play an important role in the conservation of the regional herpetofauna. The overall herpetological value of these forest fragments (Belluardo et al. 2021a) also suggests that even areas impacted by high levels of human pressure and outside the national network of protected areas can host interesting and sometimes irreplaceable levels of herpetological diversity and should not be overlooked by herpetological research in order to complete the cataloguing of Malagasy reptiles.

## Supplementary Material

XML Treatment for Paragehyra tsaranoro

## ﻿Additional information

### Author ORCIDs

Francesco Belluardo https://orcid.org/0000-0002-3967-2686

Costanza Piccoli https://orcid.org/0000-0002-1234-5706

Javier Lobón-Rovira https://orcid.org/0000-0003-4380-9427

Ivo Oliveira Alves https://orcid.org/0009-0000-1187-4211

Franco Andreone https://orcid.org/0000-0001-9809-5818

Gonçalo M. Rosa https://orcid.org/0000-0002-8658-8436

Angelica Crottini https://orcid.org/0000-0002-8505-3050

### Data availability

Research data presented in this study are openly available in the Mendeley Data platform at https://doi.org/10.17632/43sdv5d9k4.1 and include the 16S phylogenetic tree in nexus format and the alignments of 16S, COI, BDNF, and POMC markers used in molecular analyses.

## Supplementary materials

Supplementary material 1 Samples analysed in this study Data type xls Explanation note Samples analysed in this study with information on sampling localities, coordinates, elevation, extraction code, field number, institutional catalogue number (for voucher specimens), status, and GenBank accession numbers for fragments of the cytochrome oxidase I gene (COI), the 3’ terminus of the 16S rRNA gene (16S), pro-opiomelanocortin gene (POMC), brain-derived neurotrophic factor gene (BDNF) and recombination activating gene 1 (Rag1). Samples of *Paragehyratsaranoro* sp. nov. and the individuals of *P.felicitae* analysed in this study for the first time are marked with an asterisk (*), while the reaming data are from Crottini et al. (2015). Rag1 accession numbers are reported (from Crottini et al. 2015), although this marker has not been included in our analyses. Accession numbers of newly generated sequences are in bold. https://binary.pensoft.net/file/1343422This dataset is made available under the Open Database License (http://opendatacommons.org/licenses/odbl/1.0/). The Open Database License (ODbL) is a license agreement intended to allow users to freely share, modify, and use this Dataset while maintaining this same freedom for others, provided that the original source and author(s) are credited.Francesco Belluardo, Costanza Piccoli, Javier Lobón-Rovira, Ivo Oliveira Alves, Malalatiana Rasoazanany, Franco Andreone, Gonçalo M. Rosa, Angelica Crottini

Supplementary material 2 Measurements and variation of non-diagnostic morphological characters of *Paragehyra* specimens Data type xls Explanation note Measurements and variation of non-diagnostic morphological characters of *Paragehyra* specimens. Specimens of *Paragehyratsaranoro* sp. nov. and the new individuals of *P.felicitae* analysed in this study are highlighted in bold, while the remaining specimens are from Crottini et al. (2015) (with the relative information on measurements and states/numbers). Refer to the Materials and methods section of the manuscript for the definitions of characters. Measurements are expressed in mm except for SLMP, 2PM, SCE, IN, SL, and PCP where symbols/numbers indicate the state or number of observed lamellae/scales/pores. Tail length (TAL) is provided in parenthesis if regenerated. The number of subdigital lamellae (SLMP) is provided for the left side of manus and pes (-, lamellae absent). SCE (+, present; –, absent). IN (#, number of internasal scales; +, presence of concave groove between nasals; –, absence of concave groove between nasals; c, nasals in contact; nc, nasals not in contact). The number of enlarged supralabial scales (SL) and precloacal pores (PCP) are provided for the right and left sides. NA, not available. https://binary.pensoft.net/file/1343423This dataset is made available under the Open Database License (http://opendatacommons.org/licenses/odbl/1.0/). The Open Database License (ODbL) is a license agreement intended to allow users to freely share, modify, and use this Dataset while maintaining this same freedom for others, provided that the original source and author(s) are credited.Francesco Belluardo, Costanza Piccoli, Javier Lobón-Rovira, Ivo Oliveira Alves, Malalatiana Rasoazanany, Franco Andreone, Gonçalo M. Rosa, Angelica Crottini
